# Causal inference for the covariance between breeding values under identity disequilibrium

**DOI:** 10.1186/s12711-022-00750-6

**Published:** 2022-09-23

**Authors:** Rodolfo J. C. Cantet, Belcy K. Angarita-Barajas, Natalia S. Forneris, Sebastián Munilla

**Affiliations:** 1 Departamento de Producción Animal, Facultad de Agronomía, Universidad de Buenos Aires, 1417 Ciudad Autónoma de Buenos Aires, Argentina; 2 Instituto de Investigaciones en Producción Animal (INPA), Consejo Nacional de Investigaciones Científicas y Técnicas (CONICET), Buenos Aires, Argentina

## Abstract

**Background:**

The covariance matrix of breeding values is at the heart of prediction methods. Prediction of breeding values can be formulated using either an “observed” or a theoretical covariance matrix, and a major argument for choosing one or the other is the reduction of the computational burden for inverting such a matrix. In this regard, covariance matrices that are derived from Markov causal models possess properties that deliver sparse inverses.

**Results:**

By using causal Markov models, we express the breeding value of an individual as a linear regression on ancestral breeding values, plus a residual term, which we call residual breeding value (RBV). The latter is a noise term that accounts for the uncertainty in prediction due to lack of fit of the linear regression. A notable property of these models is the parental Markov condition, through which the multivariate distribution of breeding values is uniquely determined by the distribution of the mutually independent RBV. Animal breeders have long been relying on a causal Markov model, while using the additive relationship matrix as the covariance matrix structure of breeding values, which is calculated assuming gametic equilibrium. However, additional covariances among breeding values arise due to identity disequilibrium, which is defined as the difference between the covariance matrix under the multi-loci probability of identity-by-descent ($\varvec{\Sigma}$) and its *expectation* under gametic phase equilibrium, i.e., **A**. The disequilibrium term $\varvec{\Sigma}$−**A** is considered in the model for predicting breeding values called the “ancestral regression” (AR), a causal Markov model. Here, we introduce the “ancestral regression to parents” (PAR) causal Markov model, which reduces the computational burden of the AR approach. By taking advantage of the conditional independence property of the PAR Markov model, we derive covariances between the breeding values of grandparents and grand-offspring and between parents and offspring. In addition, we obtain analytical expressions for the covariance between collateral relatives under the PAR model, as well as for the inbreeding coefficient.

**Conclusions:**

We introduced the causal PAR Markov model that captures identity disequilibrium in the covariances among breeding values and produces a sparse inverse covariance matrix to build and solve a set of mixed model equations.

**Supplementary Information:**

The online version contains supplementary material available at 10.1186/s12711-022-00750-6.

## Background

In genetic evaluations, the best linear unbiased predictor (BLUP, [1]) is used to predict the breeding values (BV) of selection candidates. Breeding values are a presumed compound random variable that arises from the effect of a large number of genes in gametic equilibrium and follows a multivariate normal distribution across the population [1–3]. Prediction of BV can be formulated using either an “observed” or a theoretical covariance matrix of BV. The latter model takes advantage from the parental Markov condition of the covariance between BV of the parents and their offspring [4] and theorem 1.2.7 in [5]. Throughout this paper, we will refer to these models as recursive causal Markov models or Markov models for short.

In Markov models, the BV of an individual is expressed as a linear regression on parental, or parental and grandparental BV, plus a residual breeding value (RBV) term. RBV is a noise term that accounts for the lack of fit of the regression with respect to the statistical information that the ancestors convey to predict the BV of an individual. The parental Markov condition implies that the multivariate distribution of BV is uniquely determined by the distribution of these RBV, which are mutually independent random variables. This is a familiar property of a Markov model well-known to animal breeders: the regular Animal Model [1] where the RBV are termed “Mendelian sampling terms”.

The theoretical covariance matrix of a Markov model describes the sequences of BV across generations as a stochastic process, which is represented by the regression of the BV of an individual on ancestral BV [1, 6]. Starting with the additive relationship matrix as the covariance structure for BV under the regular Animal Model, more informative and realistic Markov models should account for the different proportions of the genomes from the grandparents in the gametes that form the individual, which originate at meiosis due to recombination. We will show that this can be accomplished by extending the concept of identity disequilibrium of Weir and Cockerham [7]. Identity disequilibrium is accounted for in the ancestral regression (AR) Markov model of Cantet et al. [8].

In the original presentation of the AR model, we came short of deriving the covariances between the BV of relatives. Therefore, our objective is threefold. The first goal is to present a causal Markov model that we refer to as the ancestral regression to parents (PAR) and that simplifies inversion of the covariance matrix of BV when the AR model is correct. The second objective is to give explicit expressions for the covariances between the BV of an animal with those of its grandparents and parents under identity disequilibrium. Finally, we derive the covariance of the BV of an individual with its collaterals (full and half-sibs) assuming that PAR is correct. These latter covariances are needed for genomic prediction of animals without an own record and without progeny. However, issues with parameter estimation and how to specifically use the AR and PAR models for genomic prediction will be pursued elsewhere. In the three additional files of this paper, we describe in detail some theoretical arguments and all the derivations.

## Methods

### Covariance between breeding values under segmental inheritance

The covariance matrix of breeding values (BV) has traditionally been calculated by conditioning on relatedness [1]. In this section, we extend the definition of genetic relationships at one locus to the multilocus situation with segmental inheritance and obtain the parameters that enter into the covariance of BV from the recursive regression of the BV of an animal on the parental and grandparental breeding values [8].

### Covariance between BV under gametic phase equilibrium

Under the assumption of gametic phase equilibrium and for any two animals X and Y that share one or more common ancestors, Cockerham [9], Kempthorne [10], and Harris [11] obtained the expression:

1 $${\text{cov}}\left({a}_{\text{X}}, {a}_{\text{Y}}\right)={\text{A}}_{\text{XY}}{\upsigma}_{\text{A}}^{2},$$

where a_X_ and $a_{\text {Y}}$ stand for the BV of ${\text{X}}$ and ${\text{Y}}$, ${\text{A}}_{\text{XY}}$ is the additive genetic relationship between ${\text{X}}$ and ${\text{Y}}$, and ${\upsigma}_{\text{A}}^{2}$ is the additive variance. The additive genetic relationship is related to probabilities of identity-by-descent (IBD, [12]) and thus ${\text{cov}}\left({a}_{\text{X}}, {a}_{\text{Y}}\right)$ in Eq. (1) is always greater or equal to zero. In other words, related individuals ${\text{X}}$ and ${\text{Y}}$ have a positive covariance that is due to a non-zero probability of sharing genes that are IBD. On the other hand, a zero covariance indicates that ${\text{X}}$ and ${\text{Y}}$ are unrelated.

In addition, the fact that $\text{cov}({a}_{\text{X}},{a}_{\text{Y}}$) is ≥ 0 explains a joint behavior of the breeding values of $\text{X}$ and $\text{Y}$ by which they are likely to simultaneously take large (or small) values. As a result, the non-negative covariance property ensures that animals with larger BV than the mean will tend to have progeny with larger BV than the mean, whereas animals with smaller BV than the mean will produce progeny that tend to have smaller BV than the mean. Consequently, $\text{cov}({a}_{\text{X}},{a}_{\text{Y}}$) ≥ 0 ensures the success of selection in either direction when employing predictions of BV calculated under such a premise. This useful stochastic behavior is known as “positive quadrant dependence” [13] and we seek to extend this property to multilocus distributions of IBD.

Under gametic phase equilibrium, the additive relationship ${\text{A}}_{\text{XY}}$ in Eq. (1) is equal to twice the coefficient of coancestry between $\text{X}$ and $\text{Y}$ [12], which in turn is equal to the probability that one random sampled allele in any locus in the genome of $\text{X}$ is IBD to a random sampled allele at the same locus but in individual $\text{Y}$; this probability is denoted as $P(\text{X}\equiv \text{Y})$. As a measure of relatedness between two animals, the coefficient of coancestry is then a probability of IBD at a single locus, which is the same for any autosomal loci under the assumption of gametic phase equilibrium. The concept of IBD is always in relation to a “founder population” [14–16].

### Covariance between breeding values under segmental inheritance

The original definition of IBD alludes to a discrete random variable at a single locus. However, the genome is inherited in segments produced by recombination during meiosis [15], which introduces dependence among multiple loci. When calculating the covariance matrix of BV, the assumption of independence of causal locus effects is challenged by physical dependence due to closeness, i.e., linkage, and pervasive dependence among non-allelic loci [17] due to population structure [18] and/or selection [19]. Therefore, the genotypic frequencies may deviate from their expectation under a scenario of random union of gametes [20], a phenomenon that is called gametic phase disequilibrium. Recombination that occurs during meiosis breaks these associations among non-allelic loci and produces segments of DNA that are carried by descendant animals after several generations [15, 17].

Donnelly [21] proposed a “continuous IBD” model to calculate the probability that individuals in a given relationship share any part of their genomes IBD. Their proposal takes recombination into account and serves to model inheritance of segments [15]. After [21], Guo [22] defined the proportion of genome that is shared IBD by two individuals as the sum of all the chromosome segments that are IBD at the same chromosome loci. However, for this relationship to comply with Eq. (1) and to produce a proper covariance matrix of BV that is inversible and Markovian, some theoretical considerations should be met.

First, we need a generalization of the definition of pointwise probabilities of IBD to segments. Nolan [23] defined the probability of IBD for shared segments through a multivariate binary random variable as follows. Let $j$ be a position on chromosome $k$ such that $j=1, \dots , n$. Also let ${i}_{j}$ be a binary random variable that is equal to 1 if a recombination occurred between positions $j$ and $j+1$ on the chromosome, and equal to 0 otherwise. A given sequence (${i}_{1}, \dots , {i}_{n}$) of 0s and 1s on chromosome $k$ is called a “recombination pattern” and has a probability ${P}_{k}({i}_{1}, \dots , {i}_{n})$ attached to it. The continuous IBD random variable $\text{X}(t)$ is a binary function equal to 1 if at position $t$ the genetic material on chromosome $k$ of a pair of gametes, from either the same or from different individuals, originates in the same grandparent, whereas it is 0 otherwise. The positions in the recombination pattern ${i}_{1}, \dots , {i}_{n}$ where $\text{X}(t)$ changes from 0 to 1 or from 1 to 0 are the precise locations where a crossover has occurred [23]. The multilocus probabilities of IBD are then equal to:

2 $$P\left({\text{X}}_{1}\equiv {\text{Y}}_{1},..,{\text{X}}_{n}\equiv {\text{Y}}_{n}\right)=P\left(\text{X}\left({t}_{1}\right)={i}_{1},\text{X}\left({t}_{2}\right)-\text{X}\left({t}_{1}\right)=\left|{i}_{2}-{i}_{1}\right|, . . ,\text{X}\left({t}_{n+1}\right)-\text{X}\left({t}_{n}\right)=\left|{i}_{n+1}-{i}_{n}\right|\right).$$

Next, we use definition (2) to write a covariance matrix of BV with elements equal to the product of the multilocus probabilities of IBD, summed over chromosomes, times the additive variance:

3 $$\text{cov}\left({a}_{\text{X}}, {a}_{\text{Y}}\right)={\sum}_{k=1}^{K}{P}_{k}\left({\text{X}}_{1}\equiv {\text{Y}}_{1},..,{\text{X}}_{n}\equiv {\text{Y}}_{n}\right){\sigma}_{\text{A}}^{2}={\Sigma}_{\text{XY}}{\upsigma}_{\text{A}}^{2},$$

where $K$ is the number of chromosomes of the diploid species. The scalar ${{\Sigma}}_{\text{XY}}$ is termed “multilocus additive relationship”, and it is defined to be equal to:

4 $${{\Sigma}}_{\text{XY}} ={\sum}_{k=1}^{K}{P}_{k} \left({\text{X}}_{1}\equiv {\text{Y}}_{1},..,{\text{X}}_{n}\equiv {\text{Y}}_{n}\right).$$

Notice that the covariance in Eq. (3) is greater or equal to zero because it is a product of a sum of probabilities times a positive number, and this guarantees that BV are positive quadrant dependent [13] random variables.

If we now add and subtract ${\text{A}}_{\text{XY}}$ to ${{\Sigma}}_{\text{XY}}$ in Eq. (4) we obtain:

5 $${\Sigma}_{\text{XY}} = {{\text{A}}_{\text{XY}} +(\Sigma}_{\text{XY}} - {\text{A}}_{\text{XY}}).$$

Thus, the multilocus additive relationship is equal to the single-locus additive relationship plus a “disequilibrium” term that measures how distant is the realized relationship from ${\text{A}}_{\text{XY}}$. In the next section, we relate ${\Sigma}_{\text{XY}} - {\text{A}}_{\text{XY}}$ to the parameters of the ‘ancestral regression’ (AR) of Cantet et al. [8].

We call the disequilibrium term ${\Sigma}_{\text{XY}} - {\text{A}}_{\text{XY}}$ “identity disequilibrium” after ideas that were first discussed by Weir and Cockerham [7] and Cockerham and Weir [24]. When considering the effect of recombination on the probability of IBD of alleles at two linked loci (say 1 and 2) for animals $\text{X}$ and $\text{Y}$, Weir and Cockerham [7] attributed to JBS Haldane the following inequality:

6 $$P\left({\text{X}}_{1}\equiv {\text{Y}}_{1},{\text{X}}_{2}\equiv {\text{Y}}_{2}\right) \ge P\left({\text{X}}_{1}\equiv {\text{Y}}_{1}\right) P\left({\text{X}}_{2}\equiv {\text{Y}}_{2}\right).$$

In words, Eq. (6) says that the probability of IBD at two linked locations on a chromosome is higher or equal than the product of the marginal probabilities at each locus when $\text{X}$ and $\text{Y}$ are related$.$ The value of $P\left({\text{X}}_{1}\equiv {\text{Y}}_{1},{\text{X}}_{2}\equiv {\text{Y}}_{2}\right)$ depends on the pedigree and on the recombination rate between loci 1 and 2. Weir and Cockerham [7] called identity disequilibrium the difference between the joint probability of IBD at two loci and the product of the single locus probabilities of IBD. Formally, identity disequilibrium at two loci is equal to:

7 $$P\left({\text{X}}_{1}\equiv {\text{Y}}_{1},{\text{X}}_{2}\equiv {\text{Y}}_{2}\right)-P\left({\text{X}}_{1}\equiv {\text{Y}}_{1}\right) P\left({\text{X}}_{2}\equiv {\text{Y}}_{2}\right).$$

Identity disequilibrium is the main source for variation in actual inbreeding [7] or relationships [25]. Expression (4) in Weir et al. [26] (page 401) formalizes the link between the variance of the inbreeding coefficient at two linked loci and identity disequilibrium as displayed in Eq. (7).

### Ancestral regression

The “ancestral regression” (AR) model is a recursive causal Markov model for BV under identity disequilibrium that was developed by Cantet et al. [8]. For completeness, more details about this model are in Additional file 2. According to this model, the regression equation of the BV of $\text{X}$ on the BV of its parents and grandparents is:

8 $${a}_{\text{X}}=0.5{a}_{\text{S}}+0.5{a}_{\text{D}}+{\upbeta}_{\text{S}}\left({a}_{\text{SS}}-{a}_{\text{DS}}\right)+{\upbeta}_{\text{D}}\left({a}_{\text{SD}}-{a}_{\text{DD}}\right)+{\upphi}_{\text{X}},$$

where subscripts refer to parents ($\text{S}$ = sire and $\text{D}$ = dam) and grandparents ($\text{SS}$ = paternal grandsire; $\text{DS}$ = paternal granddam; $\text{SD}$ = maternal grandsire; $\text{DD}$ = maternal granddam) of individual $\text{X}$, and ${\upphi}_{\text{X}}$ is the residual breeding value (RBV). It is shown in  Additional file 2 that the disequilibrium term in the ancestral regression is equal to:

9 $$\begin{array}{ll}&0.5 \left({P}_{\text{S}}-0.5\right)\left({a}_{\text{SS}}-{a}_{\text{DS}}\right)+0.5 \left({P}_{\text{D}}-0.5\right)\left({a}_{\text{SD}}-{a}_{\text{DD}}\right)\\& \qquad{=\upbeta}_{\text{S}} \left({a}_{\text{SS}}-{a}_{\text{DS}}\right)+{\upbeta}_{\text{D}} \left({a}_{\text{SD}}-{a}_{\text{DD}}\right).\end{array}$$

Either side of the equalities $0.5 \left({P}_{\text{S}}-0.5\right)= {\upbeta}_{\text{S}}$ and $0.5 \left({P}_{\text{D}}-0.5\right)$ = ${\upbeta}_{\text{D}}$ expresses identity disequilibrium. In the ancestral regression, ${\upbeta}_{\text{S}}$ and ${\upbeta}_{\text{D}}$ contain the identity disequilibrium and represent the excess, or the deficiency, in the BV of the paternal and maternal grandsires, i.e., ${a}_{\text{SS}}$ and ${a}_{\text{SD}}$, with respect to the paternal and maternal granddams, i.e., ${a}_{\text{DS}}$ and ${a}_{\text{DD}}$, in the paternal and maternal gametes, respectively (see Additional file 2). On the other hand, ${\upbeta}_{\text{S}}$ and ${\upbeta}_{\text{D}}$ may be zero due to the fact that under identity equilibrium ${P}_{S}= {P}_{\text{D}}=0.5$. For standardized covariances, as used here (i.e., additive genetic variance equal to 1), ${\upbeta}_{\text{S}}$ is the path coefficient for the *contrast* ${a}_{\text{SS}}-{a}_{\text{DS}}$, while ${\upbeta}_{\text{D}}$ is that for the *contrast* ${a}_{\text{SD}}-{a}_{\text{DD}}$ (see Eq. (2) in Wright [27]). For the more general case in which the additive variance differs from 1, ${\upbeta}_{\text{S}}$ and ${\upbeta}_{\text{D}}$ are path *regressions* (see Eq. (1) in Wright [27]) or, simply, partial regression coefficients [4]. In Appendix  1, the general expression for the variance of a BV under the ancestral regression (i.e., without setting the additive genetic variance to 1) is shown to be equal to:

10 $$\text{Var}\left({a}_{\text{x}}\right)=\left\{\begin{array}{l}0.25\left[2+{F}_{\text{S}}+{F}_{\text{D}}+2{\Sigma}_{\text{S},\text{D}}\right]+{\upbeta}_{\text{S}}^{2} \left[2+{F}_{\text{SS}}+{F}_{\text{DS}}-2{\Sigma}_{\text{SS},\text{DS}}\right]\\ +{\upbeta}_{\text{D}}^{2} \left[2+{F}_{\text{SD}}+{F}_{\text{DD}}-2{\Sigma}_{\text{SD},\text{DD}}\right]+2{\upbeta}_{\text{S}}{\upbeta}_{\text{D}}\left[{\Sigma}_{\text{SS},\text{SD}}-{\Sigma}_{\text{SS},\text{DD}}-{\Sigma}_{\text{DS},\text{SD}}+{\Sigma}_{\text{DS},\text{DD}}\right]\\ +0.5{\upbeta}_{\text{S}}\left[{\Sigma}_{\text{SS},\text{S}}-{\Sigma}_{\text{DS},\text{S}}+{\Sigma}_{\text{SS},\text{D}}-{\Sigma}_{\text{DS},\text{D}}\right]+0.5{\upbeta}_{\text{D}}\left[{\Sigma}_{\text{SD},\text{S}}-{\Sigma}_{\text{DD},\text{S}}+{\Sigma}_{\text{SD},\text{D}}-{\Sigma}_{\text{DD},\text{D}}\right]\end{array}\right\}{\upsigma}_{\text{A}}^{2}+\text{Var}\left({\upphi}_{\text{X}}\right).$$

Inbreeding also increases identity disequilibrium [7, 24] and, thus, the variance of the $\Sigma$ relationships. Equation (10) is complex because the AR model involves the BV of six individuals that may be related and may also be inbred.

The inbreeding values of the grandparents and parents of $\text{X}$ that appear in Eq. (10), i.e., ${F}_{i}$ ($i$ = 1, …, 6), should not be confused with the usual measure of inbreeding of Wright [28] under gametic equilibrium. Under identity disequilibrium, the inbreeding coefficient must be redefined, and this is done in Appendix 2. The formula to calculate inbreeding under AR is:

11 $${F}_{\text{X}}=0.5\left[\begin{array}{l}{\Sigma}_{\text{SD}}+{\upbeta}_{\text{S}}\left({\Sigma}_{\text{SS},\text{D}}-{\Sigma}_{\text{DS},\text{D}}\right)+{\upbeta}_{\text{D}}\left({\Sigma}_{\text{SD},\text{S}}-{\Sigma}_{\text{DD},\text{S}}\right)\\ +{\upbeta}_{\text{S}}{\upbeta}_{\text{D}}({\Sigma}_{\text{SS},\text{SD}}-{\Sigma}_{\text{SS},\text{DD}}-{\Sigma}_{\text{DS},\text{SD}}+{\Sigma}_{\text{DS},\text{DD}})\end{array}\right].$$

If all ${F}_{i}$ in Eq. (9) are 0, the expression for $\text{Var}\left({a}_{\text{x}}\right)$ reduces to:

12 $$\text{Var}\left({a}_{\text{x}}\right)=\left[0.5+2({\upbeta}_{\text{S}}^{2}+{\upbeta}_{\text{D}}^{2})\right]{\upsigma}_{\text{A}}^{2}+\text{Var}\left({\upphi}_{\text{X}}\right).$$

For the purpose of comparison, the variance of the BV of individual $\text{X}$ under identity equilibrium is equal to $\text{Var}\left({a}_{\text{x}}\right)={0.5\upsigma}_{\text{A}}^{2}+\text{Var}\left({\upphi}_{\text{X}}^{*}\right)$, where the asterisk on ${\upphi}_{\text{X}}$ refers to the assumption of identity equilibrium. Since $2({\upbeta}_{\text{S}}^{2}+{\upbeta}_{\text{D}}^{2})\ge 0$, $\text{Var}\left({\upphi}_{\text{X}}\right)\le \text{Var}\left({\upphi}_{\text{X}}^{*}\right)$, the equality of $\text{Var}\left({\upphi}_{\text{X}}\right)$ and $\text{Var}\left({\upphi}_{\text{X}}^{*}\right)$ holds only if ${\upbeta}_{\text{S}}={\upbeta}_{\text{D}}=0$. This result was previously obtained by Cantet and Vitezica [29] for any predictor of BV that incorporates pedigree and genomic information. Pourahmadi [30] found that there is a negative relationship between the diagonal element of the inverse covariance matrix and prediction error variance of the random variable. Therefore, in general AR displays smaller variance of RBV, higher inverse diagonal elements of, reduced prediction error variance of BV, and increased accuracy of BV prediction, when compared to the regular animal model.

For a vector of BV under the AR model ordered such that the BV of any ancestor precedes those of all its descendants, we can write the causal Markov model in vector–matrix form as [31, 32]:

13 $$\mathbf{a}=\mathbf{B}\mathbf{a}+{\varvec{\upphi}}.$$

Matrix $\mathbf{B}$ is *lower triangular* because genomes always descend from ancestors to descendants. There are at most seven elements different from zero in any row of **B**: 0.5 for the columns belonging to the parents $\text{S}$ and $\text{D}$, ${\upbeta}_{\text{S}}$ for $\text{SS}$, ${-\upbeta}_{\text{S}}$ for $\text{DS}$, ${\upbeta}_{\text{D}}$ for $\text{SD}$, ${-\upbeta}_{\text{D}}$ for $\text{DD}$ and 1 for the diagonal, all other elements are zero. Working algebraically in Eq. (13) results in a triangular system of equations [32]:

14 $$\left(\mathbf{I}-\mathbf{B}\right)\mathbf{a}={\varvec{\upphi}}\Rightarrow \mathbf{a}={\left(\mathbf{I}-\mathbf{B}\right)}^{{\mathbf{-1}}}{\varvec{\upphi}}.$$

The expectation and covariance matrix of $\mathbf{a}$ in Eq. (13) are respectively equal to:

$$\text{E}\left(\mathbf{a}\right)={\left(\mathbf{I}-\mathbf{B}\right)}^{-1}\text{E}\left({\varvec{\upphi}}\right)={\mathbf{0}}$$

15 $$\text{and}\, \text{Var}\left(\mathbf{a}\right)={\varvec{\Sigma}}={\left(\mathbf{I}-\mathbf{B}\right)}^{\mathbf{-1}}\text{Var}\left({\varvec{\upphi}}\right){\left(\mathbf{I}-{\mathbf{B}}^{\mathbf{^{\prime}}}\right)}^{\mathbf{-1}}.$$

In Eq. (15), $\text{Var}({\varvec{\upphi}})$ is a diagonal matrix ($\mathbf{D}$), which implies independence among the RBV of different animals due to the parental Markov property. The inverse of the covariance matrix is:

16 $${{\varvec{\Sigma}}}^{-1}=\left(\mathbf{I}-{\mathbf{B}}^{\mathbf{^{\prime}}}\right){\mathbf{D}}^{\mathbf{-1}}\left(\mathbf{I}-\mathbf{B}\right).$$

Cantet et al. [8] proposed an algorithm to calculate ${{\varvec{\Sigma}}}^{-1}$ that was derived from the method to compute ${\mathbf{A}}^{-1}$ [33], by inferring the pattern of non-zero elements in ${{\varvec{\Sigma}}}^{-1}$ from the sparsity pattern of $\mathbf{B}$ and the diagonal elements of **D**.

### Ancestral regression to parents (PAR)

Whereas the algorithm to easily invert ${\varvec{\Sigma}}$ [8] makes it possible to build the mixed model equations to fit the AR without direct inversion of the covariance matrix of BV, the amount of memory needed is greater than that to calculate ${\mathbf{A}}^{-1}$. Let the rows of **B** related to animals $i$ and $j$ be ${\mathbf{B}}_{i}$ and ${\mathbf{B}}_{j}$, respectively, whereas let ${\text{D}}_{ii}$ be the variance of the RBV of $i$, each non-zero element of the sparse matrix ${\mathbf{B}}_{i}{\mathbf{D}}_{ii}{\mathbf{B}}_{j}^{\boldsymbol{^{\prime}}}$ is called a ‘contribution’ to the elements of ${{\varvec{\Sigma}}}^{-1}$. For a single trait model, ${{\varvec{\Sigma}}}^{-1}$ is written down in symmetric sparse format with 28 contributions per animal, compared to six contributions per animal to calculate ${\mathbf{A}}^{-1}$ [33]. In the multiple trait case ($t$ traits and $q$ animals), the number of contributions in the animal model where the covariance matrix comes from a Kronecker product of the $t\times t$ covariance matrix of BV from different traits times $\mathbf{A}$, the total number of contributions is equal to $(6t+4.5t(t-1))q$, whereas in the AR model, the number of contributions is $(28t+24.5t(t-1))q$. For the genetic evaluation of growth in beef cattle where $t=4$ and assuming that one million animals are evaluated, the number of non-zero elements in the mixed model equations is equal at least to 78 million in the animal model with ${\mathbf{A}}^{-1}$ and 406 million in the AR model, i.e., more than 5.2 times larger. Thus, with data from several million animals, fitting a multiple trait AR model may be prohibitive. Therefore, we will look for a causal model of genetic evaluation that induces a storage of non-zeros similar to the animal model and that also accounts for identity disequilibrium.

As discussed in the section of Additional file 3 entitled *A recursive calculation of the rows and columns of* ${\varvec{\Sigma}}$, the BV of an animal in the AR model is independent of the BV of any other animal when conditioning on those of its grandparents and parents. The random vector ${\mathbf{a}}_{\text{A}}^{{\prime}}=\left[{a}_{\text{SS}} {a}_{\text{DS}} {a}_{\text{SD}} {a}_{\text{DD}} {a}_{\text{S}} {a}_{\text{D}}\right]$ contains a Markov blanket [34] for estimating ${\upbeta}_{\text{S}}$ and ${\upbeta}_{\text{d}}$, i.e., a set of locally sufficient statistics for both parameters [5]. The covariance matrix of ${\mathbf{a}}_{\text{A}}$ is equal to:

17 $${{\varvec{\Sigma}}}_{\text{A}}=\left[\begin{array}{cccccc}{\Sigma}_{\text{SS},\text{SS}}& {\Sigma}_{\text{SS},\text{DS}}& {\Sigma}_{\text{SS},\text{SD}}& \left.{\Sigma}_{\text{SS},\text{DD}}\right|& {\Sigma}_{\text{SS},\text{S}}& {\Sigma}_{\text{SS},\text{D}}\\ {\Sigma}_{\text{DS},\text{SS}}& {\Sigma}_{\text{DS},\text{DS}}& {\Sigma}_{\text{DS},\text{SD}}& \left.{\Sigma}_{\text{DS},\text{DD}}\right|& {\Sigma}_{\text{DS},\text{S}}& {\Sigma}_{\text{DS},\text{D}}\\ {\Sigma}_{\text{SD},\text{SS}}& {\Sigma}_{\text{SD},\text{DS}}& {\Sigma}_{\text{SD},\text{SD}}& \left.{\Sigma}_{\text{SD},\text{DD}}\right|& {\Sigma}_{\text{SD},\text{S}}& {\Sigma}_{\text{SD},\text{D}}\\ {\Sigma}_{\text{DD},\text{SS}}& {\Sigma}_{\text{DD},\text{DS}}& {\Sigma}_{\text{DD},\text{SD}}& \left.{\Sigma}_{\text{DD},\text{DD}}\right|& {\Sigma}_{\text{DD},\text{S}}& {\Sigma}_{\text{DD},\text{D}}\\ \overline{{\Sigma}_{\text{S},\text{SS}}}& \overline{{\Sigma}_{\text{S},\text{DS}}}& \overline{{\Sigma}_{\text{S},\text{SD}}}& \left.\overline{{\Sigma}_{\text{S},\text{DD}}}\right|& \overline{{\Sigma}_{\text{S},\text{S}}}& \overline{{\Sigma}_{\text{S},\text{D}}}\\ {\Sigma}_{\text{D},\text{SS}}& {\Sigma}_{\text{D},\text{DS}}& {\Sigma}_{\text{D},\text{SD}}& \left.{\Sigma}_{\text{D},\text{DD}}\right|& {\Sigma}_{\text{D},\text{S}}& {\Sigma}_{\text{D},\text{D}}\end{array}\right]=\left[\begin{array}{cc}{{\varvec{\Sigma}}}_{\text{G}}& {{\varvec{\Sigma}}}_{\text{G},\text{P}}\\ {{\varvec{\Sigma}}}_{\text{P},\text{G}}& {{\varvec{\Sigma}}}_{\text{P}}\end{array}\right].$$

Matrix ${{\varvec{\Sigma}}}_{\text{G}}$ is 4 × 4 and contains the covariances among the BV of the grandparents, and ${{\varvec{\Sigma}}}_{\text{G},\text{P}}$ is a 4 × 2 matrix of the covariances between the grandparents and parents. They are respectively equal to:

$${{\varvec{\Sigma}}}_{\text{G}}= \left[\begin{array}{cccc}1+{F}_{\text{SS}}& {{\varvec{\Sigma}}}_{\text{SS},\text{DS}}& {{\varvec{\Sigma}}}_{\text{SS},\text{SD}}& {{\varvec{\Sigma}}}_{\text{SS},\text{DD}}\\ {{\varvec{\Sigma}}}_{\text{SS},\text{DS}}& 1+{F}_{\text{DS}}& {{\varvec{\Sigma}}}_{\text{DS},\text{SD}}& {{\varvec{\Sigma}}}_{\text{DS},\text{DD}}\\ {{\varvec{\Sigma}}}_{\text{SS},\text{SD}}& {{\varvec{\Sigma}}}_{\text{DS},\text{SD}}& 1+{F}_{\text{SD}}& {{\varvec{\Sigma}}}_{\text{SD},\text{DD}}\\ {{\varvec{\Sigma}}}_{\text{SS},\text{DD}}& {{\varvec{\Sigma}}}_{\text{DS},\text{DD}}& {{\varvec{\Sigma}}}_{\text{SD},\text{DD}}& 1+{F}_{\text{DD}}\end{array}\right],$$

18 $${\rm and} \, {\boldsymbol{\Sigma}}_{\rm P,G}=\left[\begin{array}{l}{{\varvec{\Sigma}}}_{\text{G},\text{S}}\\ {{\varvec{\Sigma}}}_{\text{G},\text{D}}\end{array}\right]=\left[\begin{array}{cccc}{{\varvec{\Sigma}}}_{\text{SS},\text{S}}& {{\varvec{\Sigma}}}_{\text{DS},\text{S}}& {{\varvec{\Sigma}}}_{\text{SD},\text{S}}& {{\varvec{\Sigma}}}_{\text{DD},\text{S}}\\ {{\varvec{\Sigma}}}_{\text{SS},\text{D}}& {{\varvec{\Sigma}}}_{\text{DS},\text{D}}& {{\varvec{\Sigma}}}_{\text{SD},\text{D}}& {{\varvec{\Sigma}}}_{\text{DD},\text{D}}\end{array}\right]$$

The vectors ${{\varvec{\Sigma}}}_{\text{G},\text{S}}$ and ${{\varvec{\Sigma}}}_{\text{G},\text{D}}$ in Eq. (18) are the rows of ${{\varvec{\Sigma}}}_{\text{P},\text{G}}$, and the covariance matrix of parental BV is equal to:

19 $${{\varvec{\Sigma}}}_{\text{P}}=\left[\begin{array}{ll}1+{F}_{\text{S}}& {{\varvec{\Sigma}}}_{\text{SD}}\\ {{\varvec{\Sigma}}}_{\text{SD}}& 1+{F}_{\text{D}}\end{array}\right].$$

We now write the 6 × 1 vector of covariances between the grandparents and parents of individual $\text{X}$ as follows:

20 $$\begin{array}{ll}{{\varvec{\Sigma}}}_{\text{AX}}^{^{\prime}}&=\left[\left.{{\varvec{\Sigma}}}_{\text{G},\text{X}}^{^{\prime}}\right|\right.\left.{{\varvec{\Sigma}}}_{\text{P},\text{X}}^{^{\prime}}\right] \\ &= {\left[{\Sigma}_{\text{SS},\text{X}} {\Sigma}_{\text{DS},\text{X}} {\Sigma}_{\text{SD},\text{X}} \left.{\Sigma}_{\text{DD},\text{X}}\right| {\Sigma}_{\text{S},\text{X}} {\Sigma}_{\text{D},\text{X}}\right]}^{^{\prime}}.\end{array}$$

The vector ${{\varvec{\Sigma}}}_{\text{G},\text{X}}$ includes the covariances between the BV of $\text{X}$ and those of the four grandparents, whereas ${{\varvec{\Sigma}}}_{\text{P},\text{X}}$ includes the covariances between the BV of $\text{X}$ and those of its parents. Finally, the vector ${\mathbf{B}}_{\text{X},r}$ of path coefficients in Eq. (17) is defined in the following manner:

21 $${\mathbf{B}}_{\text{X},r}={\left[{\upbeta}_{\text{S}}, {-\upbeta}_{\text{S}}, {\upbeta}_{\text{D}}, {-\upbeta}_{\text{D}}, 0.5, 0.5\right]}^{^{\prime}}.$$

From Expression (20) in [8], we can write vector ${{\varvec{\Sigma}}}_{\text{AX}}$ as a function of ${\mathbf{B}}_{\text{X}}$ as: ${{\varvec{\Sigma}}}_{\text{A}}{\mathbf{B}}_{\text{X},r}={{\varvec{\Sigma}}}_{\text{A},\text{X}}$, and in more detail, as:

22 $$\begin{bmatrix}{{\varvec{\Sigma}}}_{\text{G}} \quad {{\varvec{\Sigma}}}_{\text{G},\text{P}}\\ {{\varvec{\Sigma}}}_{\text{P},\text{G}} \quad {{\varvec{\Sigma}}}_{\text{P}}\end{bmatrix} {\mathbf{B}}_{\text{X},r}= \begin{bmatrix} {{\varvec{\Sigma}}}_{\text{G},\text{X}}\\ {{\varvec{\Sigma}}}_{\text{P},\text{X}} \end{bmatrix}$$

As first done in [35] to obtain the reduced animal model, we now absorb the first four equations in Eq. (22), which correspond to the grandparents, into the last two parental equations. The solution of the resulting system is the vector ${\varvec{\uprho}}$ (${{\varvec{\uprho}}}^{\mathbf{^{\prime}}}=\left[{\uprho}_{\text{S}}, {\uprho}_{\text{D}}\right]$), which contains the regression coefficients to regress the BV of $\text{X}$ on those of its parents, that is:

23 $$\begin{bmatrix}{{\varvec{\Sigma}}}_{\text{P}}{-{\varvec{\Sigma}}}_{\text{P},\text{G}}{{\varvec{\Sigma}}}_{\text{G}}^{-1}{{\varvec{\Sigma}}}_{\text{G},\text{P}}\end{bmatrix} {\varvec{\uprho}} =\begin{bmatrix}{{\varvec{\Sigma}}}_{\text{P},\text{X}}{-{\varvec{\Sigma}}}_{\text{P},\text{G}}{{\varvec{\Sigma}}}_{\text{G}}^{-1}{{\varvec{\Sigma}}}_{\text{G},\text{X}}\end{bmatrix}.$$

In scalar notation system, Eq. (23) is equal to:

24 $$\begin{bmatrix}{1+F}_{\text{S}} {-\Sigma}_{\text{S},\text{G}} \,\,{\Sigma}_{\text{G}}^{-1} {\Sigma}_{\text{G},\text{S}} \quad {\Sigma}_{\text{S},\text{D}} {-\Sigma}_{\text{S},\text{G}}\,\, {\Sigma}_{\text{G}}^{-1}\,\, {\Sigma}_{\text{G},\text{D}}\\ {\Sigma}_{\text{D},\text{S}} {-\Sigma}_{\text{D},\text{G}} \,\,{\Sigma}_{\text{G}}^{-1} \,\,{\Sigma}_{\text{G},\text{S}} \quad{1+F}_{\text{D}} {-\Sigma}_{\text{D},\text{G}} \,\,{\Sigma}_{\text{G}}^{-1} \,\,{\Sigma}_{\text{G},\text{D}} \end{bmatrix} \begin{bmatrix} {\uprho}_{\text{S}} \\{\uprho}_{\text{D}}\end{bmatrix}= \begin{bmatrix} {\Sigma}_{\text{S},\text{X}} {-\Sigma}_{\text{S},\text{G}}\,\, {\Sigma}_{\text{G}}^{-1}\,\, {\Sigma}_{\text{G},\text{X}}\\ {\Sigma}_{\text{D},\text{X}} {-\Sigma}_{\text{D},\text{G}} \,\,{\Sigma}_{\text{G}}^{-1} \,\,{\Sigma}_{\text{G},\text{X}}\end{bmatrix}$$

Thus, the ancestral regression to the parents parallels the recursion equation for BV in the animal model (i.e., a “parental regression”), but with unequal parental contributions due to distinct contributions of the grandparental BV. The equation for the PAR model is then equal to:

25 $${a}_{\text{X}}={\uprho}_{\text{S}}{a}_{\text{S}}+{\uprho}_{\text{D}}{a}_{\text{D}}+{\upphi}_{\text{P},\text{X}}.$$

The random variable ${\upphi}_{\text{P},\text{X}}$ is the RBV in the PAR model. The parameter spaces of ${\uprho}_{\text{S}}$ and ${\uprho}_{\text{D}}$ are 0.25 ≤  ${\uprho}_{\text{S}}$, ${\uprho}_{\text{D}}$ ≤ 0.75, and almost always ${\uprho}_{\text{S}}$ and ${\uprho}_{\text{D}}$ will be different from each other. Rather than the proportion of the genome of the individual that originates from a given parent, the difference between ${\uprho}_{\text{S}}$ or ${\uprho}_{\text{D}}$ with 0.50 is not a genome proportion but the amount of statistical information on their BV that the sire and the dam pass to a particular progeny under identity disequilibrium, instead of 0.5.

The variance of the BV in PAR is as follows:

26 $${\text{Var}}_{\uprho}\left({a}_{\text{X}}\right)={\text{Var}}_{\uprho}\left({\uprho}_{\text{S}}{a}_{\text{S}}+{\uprho}_{\text{D}}{a}_{\text{d}}\right)+{\text{Var}}_{\uprho}\left({\upphi}_{\text{P}i}\right),$$

where ${\upphi}_{\text{P}i}$ is the RBV under the PAR model for animal $i$. The subscript ρ in the variance operator or in ${{\varvec{\Sigma}}}_{\uprho}$ emphasizes the fact that the variance is calculated under the PAR. Elements of ${\varvec{\Sigma}}$ and ${{\varvec{\Sigma}}}_{\uprho}$ are different because AR and PAR are not equivalent models in the sense of [36] (see Appendix  4). Using variance operator rules, Eq. (26) results in being equal to:

27 $$\begin{aligned} {\text{Var}}_{\uprho}\left({a}_{\text{X}}\right)& ={\uprho}_{\text{S}}^{2}{\text{Var}}_{\uprho}\left[{a}_{\text{S}}\right]+{\uprho}_{\text{D}}^{2}{\text{ Var}}_{\uprho}\left[{a}_{\text{D}}\right]+2 {{\uprho}_{\text{S}}}{{\uprho}_{\text{D}}} {\text{cov}}_{\uprho}\left[{a}_{\text{S}},{a}_{\text{D}}\right]+{\text{Var}}_{\uprho}\left({\upphi}_{\text{P}i}\right)\\&={\uprho}_{\text{S}}^{2} \left(1+{F}_{\text{S}}\right)+{\uprho}_{\text{D}}^{2} \left(1+{F}_{\text{D}}\right)+2 {{\uprho}_{\text{S}}}{{\uprho}_{\text{D}}} {\Sigma}_{{\uprho} {\text{S}},\text{D}}+{\text{Var}}_{\uprho}\left({\upphi}_{\text{P}i}\right).\end{aligned}$$

Finally, we obtain the variance of the RBV by solving Eq. (27) for ${\text{Var}}_{\uprho}({\upphi}_{\text{P}i})$ as:

28 $${\text{Var}}_{\uprho}\left({\upphi}_{\text{P}i}\right)=1+{F}_{\text{X}}-\left[{\uprho}_{\text{S}}^{2}\left(1+{F}_{\text{S}}\right)+{\uprho}_{\text{D}}^{2}\left(1+{F}_{\text{D}}\right)+2{\uprho}_{\text{S}} {\uprho}_{\text{D}} {\Sigma}_{{\uprho {\text{S}}},\text{D}}\right],$$

where ${\Sigma}_{{\uprho {\text{S}}},\text{D}}$ is the covariance between the BV of the parents of $\text{X}$ under the PAR model.

As the PAR model displays the same set of conditional independencies as the animal model [1], the number of non-zeros in the coefficient matrix of the mixed model equations under PAR is equal to that in the animal model that is generated with pedigree to calculate ${\mathbf{A}}^{-1}$. To obtain predictions of BV using Henderson’s mixed model equations [1], the inverse covariance structure of BV under PAR, ${{\varvec{\Sigma}}}_{\uprho}^{-1}$, can be calculated by simply modifying the algorithm of Henderson [33] for ${\mathbf{A}}^{-1}$ without ever calculating ${{\varvec{\Sigma}}}_{\uprho}$, by replacing the 0.5 values in the contributions to ${\mathbf{A}}^{-1}$ by ${\uprho}_{\text{S}}$ or ${\uprho}_{\text{D}}$ and 0.25 values by the product of ${\uprho}_{\text{S}}$ and ${\uprho}_{\text{D}}$, or the squared values of these path coefficients.

### Covariance between the breeding values of an ancestor and a descendant in causal Markov models

For any causal Markov model, the covariance between the BV of an ancestor (${a}_{\text{A}}$) and the BV of a descendant (${a}_{\text{X}}$) results from the recursion of ${a}_{\text{X}}$ to a linear combination of BV of direct ancestors, until reaching the BV of the ancestor. At that point, $\text{cov}({a}_{\text{A}},$ ${a}_{\text{A}})=\text{V}$ ar(${a}_{\text{A}}$) and the recursion ends. Calculating this covariance is then similar to calculating additive relationships using the tabular method [37], for which individuals in a pedigree are ordered by date of birth to ensure that a descendant is never placed before a direct ancestor. The correctness of the algebraic procedure for the tabular method was demonstrated in a paper from Rohan Fernando’s group back in the 1990s [38].

### The ancestor–descendant covariance of breeding values in the ancestral regression model

As explained in the above paragraph, the ancestor–descendant covariance of BV results from the recursion of the BV of the descendant only, because of the parental Markov property. By definition:

$${\Sigma}_{\text{A},\text{X}}=\text{cov}\left({a}_{\text{A}},{a}_{\text{X}}\right).$$

Now we replace the BV of the descendant with the AR expression for the BV of $\text{X}$ in Eq. (8), as follows:

$$\begin{array}{ll}\text{cov}\left({a}_{\text{A}},{a}_{\text{X}}\right)&=\,\text{cov}\left({a}_{\text{A}},0.5{a}_{\text{S}}+0.5{a}_{\text{D}}+{\upbeta}_{\text{S}}\left({a}_{\text{SS}}-{a}_{\text{DS}}\right)+{\upbeta}_{\text{D}}\left({a}_{\text{SD}}-{a}_{\text{DD}}\right)\right) \\ &=0.5\text{cov}\left({a}_{\text{A}},{a}_{\text{S}}\right) +0.5\text{cov}\left({a}_{\text{A}},{a}_{\text{D}}\right) {+\upbeta}_{\text{S}}\left[\text{cov}\left({a}_{\text{A}},{a}_{\text{SS}}\right)-\text{cov}({a}_{\text{A}},{a}_{\text{DS}})\right]+{\upbeta}_{\text{D}}\left[\text{cov}\left({a}_{\text{A}},{a}_{\text{SD}}\right)-\text{cov}({a}_{\text{A}},{a}_{\text{DD}})\right].\end{array}$$

Because the covariance between the BV of animals $j$ and $k$ in AR is defined to be equal to $\text{cov}\left({a}_{j},{a}_{k}\right)= {\Sigma}_{j,k},$ i.e., the elements of ${\varvec{\Sigma}}$, the above equation results in being equal to:

29 $$\begin{array}{ll}{\Sigma}_{\text{A},\text{X}}=0.5\left({\Sigma}_{\text{A},\text{S}}+{\Sigma}_{\text{A},\text{D}}\right){+\upbeta}_{\text{S}}\left({\Sigma}_{\text{A},\text{SS}}-{\Sigma}_{\text{A},\text{DS}}\right)\\ \qquad \qquad {+\,\upbeta}_{\text{D}}\left({\Sigma}_{\text{A},\text{SD}}-{\Sigma}_{\text{A},\text{DD}}\right).\end{array}$$

The covariance between the BV of a sire and an offspring (say $\text{X}$) is obtained after setting the ancestor to the sire in Eq. (29), i.e., $\text{A}=\text{S}$, such that:

$$\begin{array}{ll}{\Sigma}_{\text{S},\text{X}}=0.5\left({\Sigma}_{\text{S},\text{S}}+{\Sigma}_{\text{S},\text{D}}\right){+\upbeta}_{\text{S}}\left({\Sigma}_{\text{S},\text{SS}}-{\Sigma}_{\text{S},\text{DS}}\right)\\ \qquad \qquad {+\,\upbeta}_{\text{D}}\left({\Sigma}_{\text{S},\text{SD}}-{\Sigma}_{\text{S},\text{DD}}\right).\end{array}$$

Now, since ${\Sigma}_{\text{S},\text{S}}$ is the variance of the BV of the sire and is equal to ${\Sigma}_{\text{S},\text{S}}=1+{F}_{\text{S}}$, the sire – offspring covariance of BV is equal to:

30 $$\begin{array}{ll}{\Sigma}_{\text{S},\text{X}}=0.5\left(1+{F}_{\text{S}}+{\Sigma}_{\text{S},\text{D}}\right){+\upbeta}_{\text{S}}\left({\Sigma}_{\text{SS},\text{S}}-{\Sigma}_{\text{DS},\text{S}}\right) \\ \qquad \qquad {+\upbeta}_{\text{D}}\left({\Sigma}_{\text{SD},\text{S}}-{\Sigma}_{\text{DD},\text{S}}\right).\end{array}$$

In case of gametic equilibrium, ${\upbeta}_{\text{S}}={\upbeta}_{\text{D}}=0$ and ${\Sigma}_{\text{S},\text{X}}=0.5\left(1+{F}_{\text{S}}+{2F}_{\text{X}}\right)={\text{A}}_{\text{S},\text{X}}$, as for Expression (7.4) in [38].

By a similar reasoning, the covariance between the BV of $\text{X}$ and of its dam is equal to:

31 $${\Sigma}_{\text{D},\text{X}}=\,0.5\left(1+{F}_{\text{D}}+{\Sigma}_{\text{S},\text{D}}\right) {+\upbeta}_{\text{S}}\left({\Sigma}_{\text{SS},\text{D}}-{\Sigma}_{\text{DS},\text{D}}\right){+\upbeta}_{\text{D}}\left({\Sigma}_{\text{SD},\text{D}}-{\Sigma}_{\text{DD},\text{D}}\right).$$

If the paternal grandsire is the ancestor in Eq. (31), $\text{cov}({a}_{\text{SS}},{a}_\text{X})$ under the AR model becomes equal to:

32 $${\Sigma}_{\text{SS},\text{X}}=0.5{\Sigma}_{\text{SS},\text{S}}+0.5{\Sigma}_{\text{SS},\text{D}}{+\upbeta}_{\text{S}}\left(1+{F}_{\text{SS}}-{\Sigma}_{\text{SS},\text{DS}}\right)+{\upbeta}_{\text{D}}\left({\Sigma}_{\text{SS},\text{SD}}-{\Sigma}_{\text{SS},\text{DD}}\right).$$

In the absence of inbreeding, ${\Sigma}_{\text{SS},\text{D}}={F}_{\text{SS}}={\Sigma}_{\text{SS},\text{DS}}={\Sigma}_{\text{SS},\text{SD}}={\Sigma}_{\text{SS},\text{DD}}=0$, and with ${\Sigma}_{\text{SS},\text{S}}=0.5$, the covariance between the BV of a grandsire and grand-offspring is equal to:

33 $${\Sigma}_{\text{SS},\text{X}}=0.25+{\upbeta}_{\text{S}}.$$

Under gametic equilibrium, ${\upbeta}_{\text{S}}=0$ and ${\Sigma}_{\text{SS},\text{X}}=0.25$.

Similarly, the covariance between the BV of the paternal granddam and of $\text{X}$ is:

34 $${\Sigma}_{\text{DS},\text{X}}=0.5{\Sigma}_{\text{DS},\text{S}}+0.5{\Sigma}_{\text{DS},\text{D}}{-\upbeta}_{\text{S}}\left(1+{F}_{\text{DS}}-{\Sigma}_{\text{SS},\text{DS}}\right)+{\upbeta}_{\text{D}}\left({\Sigma}_{\text{DS},\text{SD}}-{\Sigma}_{\text{DS},\text{DD}}\right).$$

If there is no inbreeding, Eq. (34) becomes equal to:

35 $${\Sigma}_{\text{DS},\text{X}}=0.25{-\upbeta}_{\text{S}}.$$

From Eqs. (33) and (35), it is deduced that $-0.25{\le\upbeta}_{\text{S}}\le 0.25$. As with the regular animal model and in the absence of inbreeding, the sum of the covariances between the grandsire and $\text{X}$ and the granddam and $\text{X}$ in AR give the parent–offspring covariance. To see this, add Eq. (33) to Eq. (35) to obtain ${\Sigma}_{\text{SS},\text{X}} + {\Sigma}_{\text{DS},\text{X}}=0.25+{\upbeta}_{\text{S}}+0.25{-\upbeta}_{\text{S}}=0.5$, which is precisely the value of the covariance between the BV of parent and offspring in the absence of inbreeding. As the values of ${\upbeta}_{\text{S}}$ and ${\upbeta}_{\text{D}}$ are set arbitrarily to the positive sign for the grandsire and the covariance parent–offspring is the same under identity equilibrium or gametic disequilibrium, it follows that the resemblance gained (or lost) by the grandsire with respect to the expected value of 0.25 under gametic equilibrium is lost (or gained) by the granddam.

The covariances of the BV of the maternal grandsire and the maternal granddam with the BV of $\text{X}$ are, respectively, equal to:

36 $${\Sigma}_{\text{SD},\text{X}}=0.5{\Sigma}_{\text{SD},\text{S}}+0.5{\Sigma}_{\text{SD},\text{D}}{+\upbeta}_{\text{S}}\left({\Sigma}_{\text{SD},\text{SS}}-{\Sigma}_{\text{SD},\text{DS}}\right)+{\upbeta}_{\text{D}}\left(1+{F}_{\text{SD}}-{\Sigma}_{\text{SD},\text{DD}}\right),$$

and

37 $${\Sigma}_{\text{DD},\text{X}}=0.5{\Sigma}_{\text{DD},\text{S}}+0.5{\Sigma}_{\text{DD},\text{D}}{+\upbeta}_{\text{S}}\left({\Sigma}_{\text{DD},\text{SS}}-{\Sigma}_{\text{DD},\text{DS}}\right)-{\upbeta}_{\text{D}}\left(1+{F}_{\text{DD}}-{\Sigma}_{\text{SD},\text{DD}}\right).$$

Similar expressions to Eqs. (36) and (37) but in the absence of inbreeding are:

$${\Sigma}_{\text{SD},\text{X}}=0.25+{\upbeta}_{\text{D}},$$

38 $$\text{and}\, {\Sigma}_{\text{DD},\text{X}}=0.25-{\upbeta}_{\text{D}}.$$

### Covariances between breeding values when relatives are not ancestors of each other in Markov causal models

Two related individuals $\text{X}$ and $\text{Y}$ that do not descend from each other have one or more common ancestors ${\text{CA}}_{1}$, …, ${\text{CA}}_{I}$, where $I$ is the number of common ancestors. A *trek* is the path between the BV of $\text{X}$ and $\text{Y}$ passing through the BV of the common ancestor ${\text{CA}}_{i}$ and is represented by ${a}_{\text{X}}\leftarrow\cdots \leftarrow{a}_{{\text{CA}}_{i}}\rightarrow\cdots \rightarrow{a}_{\text{Y}}$ (see page 12 in [4] and Expression (3.1) in [39]). Each trek contributes a value that is equal to the product of all single path coefficients involved in the trek times the variance of the BV of the common ancestor. Then, the covariance between the BV of $\text{X}$ and $\text{Y}$ can be calculated by summing contributions over all possible treks [40]:

39 $$\text{cov}\left({a}_{\text{X}}, {a}_{\text{Y}}\right)=\sum_{i=1}^{I}\left({b}_{j}\dots {b}_{{j}^{^{\prime}}}\right)\text{Var}\left({a}_{{\text{CA}}_{i}}\right),$$

where ${b}_{j}$ stands for the single path coefficient of the $j$-th ancestor in the trek. Note that these path coefficients take either the value 0.5 or $\upbeta$. In the parental regression model, all path coefficients are equal to 0.5.

A difficulty with the AR model is that the covariance between the BV of two relatives, none of which is an ancestor of the other, involves the calculation of up to 36 covariances among possibly 12 different animals (see the derivation in Appendix 3). The resulting expression is formidable and suggests the need for a simpler method to calculate covariances between collateral individuals, which is what we present in the next section.

### Covariance between breeding values under the ancestral regression model to parents when neither animal is an ancestor of each other

Once parameters $\uprho$ are estimated using Eq. (24), the genetic covariances under the PAR model are calculated by a recursion to the youngest individual, in the following manner:

40 $${\Sigma}_{{\rho {\rm A}},\text{X}}={\uprho}_{\text{S}}\boldsymbol{}{\Sigma}_{{\rho {\rm A}},\text{S}}+{\uprho}_{\text{D}}{\Sigma}_{{\rho {\rm A}},\text{D}}.$$

The subscript $\uprho$ in the elements of the covariance matrix indicates that those elements are defined under PAR. In Appendix 4, we explain the reason why AR and PAR are not Markov equivalent models and display an example in which the covariances between the BV of parent and offspring under AR and PAR are numerically different.

When neither individual is an ancestor of the other, the covariance of the BV of relatives under PAR results from replacing BV by the PAR model for breeding values:

$$\begin{array}{ll}{\text{cov}}_{\uprho}\left({a}_{\text{X}}, {a}_{\text{Y}}\right)={\text{cov}}_{\uprho}\left({\uprho}_{{\text{S}}_{\text{X}}}{a}_{{\text{S}}_{\text{X}}}+{\uprho}_{{\text{D}}_{\text{X}}}{a}_{{\text{D}}_{\text{X}}}, {\uprho}_{{\text{S}}_{\text{Y}}}{a}_{{\text{S}}_{\text{Y}}}+{\uprho}_{{\text{D}}_{\text{Y}}}{a}_{{\text{D}}_{\text{Y}}}\right) \\ \quad={\text{cov}}_{\uprho}\left({\uprho}_{{\text{S}}_{\text{X}}}{a}_{{\text{S}}_{\text{X}}}, {\uprho}_{{\text{S}}_{\text{Y}}}{a}_{{\text{S}}_{\text{Y}}}\right)+{\text{cov}}_{\uprho}\left({\uprho}_{{\text{S}}_{\text{X}}}{a}_{{\text{S}}_{\text{X}}}, {\uprho}_{{\text{D}}_{\text{Y}}}{a}_{{\text{D}}_{\text{Y}}}\right)+{\text{cov}}_{\uprho}\left({\uprho}_{{\text{D}}_{\text{X}}}{a}_{{\text{D}}_{\text{X}}}, {\uprho}_{{\text{S}}_{\text{Y}}}{a}_{{\text{S}}_{\text{Y}}}\right)\\ \quad+\,{\text{cov}}_{\uprho}\left({\uprho}_{{\text{D}}_{\text{X}}}{a}_{{\text{D}}_{\text{X}}}, {\uprho}_{{\text{D}}_{\text{Y}}}{a}_{{\text{D}}_{\text{Y}}}\right).\end{array}$$

Then, the resulting covariance between BV is:

41 $$\begin{array}{ll}{\text{cov}}_{\uprho}\left({a}_{\text{X}}, {a}_{\text{Y}}\right)={\uprho}_{{\text{S}}_{\text{X}}}{\uprho}_{{\text{S}}_{\text{Y}}}{\text{ cov}}_{\uprho}\left({a}_{{\text{S}}_{\text{X}}}, {a}_{{\text{S}}_{\text{Y}}}\right)+{\uprho}_{{\text{S}}_{\text{X}}}{\uprho}_{{\text{D}}_{\text{Y}}}{\text{ cov}}_{\uprho}\left({a}_{{\text{S}}_{\text{X}}}, {a}_{{\text{D}}_{\text{Y}}}\right)\\ \quad +\,{\uprho}_{{\text{D}}_{\text{X}}}{\uprho}_{{\text{S}}_{\text{Y}}}{\text{ cov}}_{\uprho}\left({a}_{{\text{D}}_{\text{X}}}, {a}_{{\text{S}}_{\text{Y}}}\right) +{\uprho}_{{\text{D}}_{\text{X}}}{\uprho}_{{\text{D}}_{\text{Y}}}{\text{ cov}}_{\uprho}\left({a}_{{\text{D}}_{\text{X}}}, {a}_{{\text{D}}_{\text{Y}}}\right).\end{array}$$

In general, we can write the covariance terms in Eq. (41) using Eq. (39) as follows: 

$${\text{cov}}_{\uprho}\left({a}_{{\text{S}}_{\text{X}}}, {a}_{{\text{S}}_{\text{Y}}}\right)=\left(1+{F}_{{\rho {\rm S}}}\right) {\upsigma}_{\text{A}}^{2},$$

$${\text{cov}}_{\uprho}\left({a}_{{\text{S}}_{\text{X}}}, {a}_{{\text{D}}_{\text{Y}}}\right)={\text{cov}}_{\uprho}\left({a}_{{\text{D}}_{\text{X}}}, {a}_{{\text{S}}_{\text{Y}}}\right)={\Sigma}_{{\rho {\rm SD}}}\boldsymbol{}{\upsigma}_{\text{A}}^{2},$$

$${\text{cov}}_{\uprho}\left({a}_{{\text{D}}_{\text{X}}}, {a}_{{\text{D}}_{\text{Y}}}\right)=\left(1+{F}_{{\rho {\rm D}}}\right){\upsigma}_{\text{A}}^{2}.$$

Specific expressions for the covariance between BV of full-sibs ($FS$), paternal half-sibs ($PHS$), and maternal half-sibs ($MHS$) are calculated by replacing in Eq. (41) with appropriate elements of ${\varvec{\Sigma}}$. The respective covariances between BV of $FS$, $PHS$, and $MHS$ are:

42 $${\text{cov}}_{\uprho}\left(FS\right)=\left[{\uprho}_{{\text{S}}_{\text{X}}}{\uprho}_{{\text{S}}_{\text{Y}}}\left(1+{F}_{{\rho {\rm S}}}\right)+\left[{\uprho}_{{\text{S}}_{\text{X}}}{\uprho}_{{\text{D}}_{\text{Y}}}+{\uprho}_{{\text{D}}_{\text{X}}}{\uprho}_{{\text{S}}_{\text{Y}}}\right]{\Sigma}_{{\rho {\rm SD}}}+{\uprho}_{{\text{D}}_{\text{X}}}{\uprho}_{{\text{D}}_{\text{Y}}}\left(1+{F}_{{\rho {\rm D}}}\right)\right]{\upsigma}_{\text{A}}^{2}$$

43 $${\text{cov}}_{\uprho} (PHS)=\left[{\uprho_{{\text{S}}_{\text{X}}}}{\uprho_{{\text{S}}_{\text{Y}}}} (1+{F}_{{\rho{\rm S}}})+{\uprho_{{\text{S}}_{\text{X}}}}{\uprho_{{\text{D}}_{\text{Y}}}}{\Sigma_{\uprho {{\text{S}}_{\text{X}}}{{\text{D}}_{\text{Y}}}}}+{\uprho_{{\text{S}}_{\text{Y}}}}{\uprho_{{\text{D}}_{\text{X}}}}{\Sigma_{\uprho {{\text{S}}_{\text{Y}}}{{\text{D}}_{\text{X}}}}}\right]{\upsigma}_{\text{A}}^{2}$$

44 $${\text{cov}}_{{\rm P}\rho} (MHS)=\left[{\uprho_{{\text{S}}_{\text{X}}}}{\uprho_{{\text{D}}_{\text{Y}}}}{\Sigma_{\uprho {{\text{S}}_{\text{X}}}{{\text{D}}_{\text{Y}}}}}+{{\uprho}_{{\text{S}}_{\text{Y}}}}{{\uprho}_{{\text{D}}_{\text{X}}}}{\Sigma}_{\uprho {{\text{S}}_{\text{Y}}}{{\text{D}}_{\text{X}}}}+{{\uprho}_{{\text{D}}_{\text{X}}}}{{\uprho}_{{\text{D}}_{\text{Y}}}}\left(1+{F}_{{\rho {\rm D}}}\right)\right]{\upsigma}_{\text{A}}^{2}$$

## Discussion

In this paper, we introduce the ancestral regression to parents (PAR) model, which derives from the ancestral regression model (AR) introduced by Cantet et al. [8] and show how to calculate additive covariances between the breeding values of related individuals under identity disequilibrium. Both, the AR and PAR models, and the standard parental regression model, are recursive causal Markov models [4, 5] under a multivariate Gaussian distribution of BV. The expressions for the covariances between the BV of relatives are derived using the recursive causal framework. In particular, the covariances between the BV of grandparents and grand-offspring, between parents and offspring, and the covariance between collaterals for the AR and PAR models. These covariances are different from the usual additive covariances under gametic equilibrium because they account for identity disequilibrium [7]: the difference between the realized relationship and its expected value under gametic equilibrium, which is originated in the individual processes of whole genome recombination during the formation of the gametes that originated both individuals in the relationship. These recombinations involve the genomes of the grandsire and the granddam during meiosis, and result in unique gametes that form each individual.

Causal Markov models for BV possess the property that their residual terms (referred to as RBV) are independent, thus they allow for a recursive calculation of the covariance between parent and offspring. In Markov causal models, this covariance is always positive and, in general, the covariance between the BV of any two animals in a pedigree is non-negative and, therefore, BV are positive quadrant dependent [13] random variables. Moreover, the covariance matrix of BV under PAR can be inverted with a computational effort that is proportional to the number of animals and, particularly, the inversion of the covariance matrix under PAR is similar to inverting $\mathbf{A}$ [33], but with the paternal and maternal path coefficients $\uprho$ of PAR replacing the 0.5 in the parental contributions.

While in the pre-genomic era covariances between BV of relatives were calculated on the basis of the degree of *relatedness* between animals [1] using only information from the pedigree, in recent times, animal breeders have also used dense sets of SNPs. The use of pedigree relationships is compatible with a stochastic process that mimics a “regression” to the BV of equally contributing parents, whereas genomic relationships result from using sample statistics, specifically marker frequencies, to build an observed “empirical genetic relatedness matrix” (see VanRaden [41]). Although BLUP of BV can be obtained by using either a theoretically-derived or an observed covariance matrix, the variance of the elements of the latter may be quite large. For example, Garcia-Baccino et al. [42] found that the variance of half-sib relationships among crossbred pigs was half as large when relatedness was measured in IBD segments compared to using single-loci relatedness based on the method of Han and Abney [43], and almost one fourth of the variance of “identity-by-state” calculated with the method described in [41]. In addition, and for the same crossbred population and for either ancestor–descendant relationships or for relationships of collateral relatives, Forneris et al. [44] found that the variance of relatedness measures was smaller when conditioning on pedigree and SNPs than when conditioning on markers only. More importantly, observed probabilities of IBD result in non-zero covariances between RBV [45] and, neither the parent–offspring covariance of BV does not have the parental Markov property, and the resulting covariance matrix of BV does not admit a Cholesky-root-free decomposition such as in Eq. (15).

When considering the transmission of DNA across generations in a lineage or a pedigree (see Additional file 1), Matsen and Evans [46] differentiated the genealogical or ancestry process from the differential recombination of the grandparental genomes when producing the parental gamete that produces their grand progeny. Accounting for recombination, the fraction of the additive variance that is explained by the regression of the BV of a progeny on the BV of parents and grandparents exceeds the genetic variance explained by the “parental regression” by ${\upbeta}_{\text{S}}\left({a}_{\text{SS}}-{a}_{\text{DS}}\right)+{\upbeta}_{\text{D}}({a}_{\text{SD}}-{a}_{\text{DD}})$, such that the difference of variances for the RBV in the animal model with the one from the AR is non-negative, i.e., $\text{Var}\left({\upphi}^{*}\right)-\text{Var}({\upphi}_{\text{AR}}) \ge 0$, as proven in [29]. In general, identity disequilibrium can be expressed as the matrix difference (${{\varvec{\Sigma}}}-\mathbf{A})$. When the matrix (${{\varvec{\Sigma}}}-\mathbf{A})$ is positive definite, it is possible to recover more additive variance and thus, heritability, by using our covariance matrices ${\varvec{\Sigma}}$ (of AR) or ${\varvec{\Sigma}}$_ρ_ (of PAR) in the resulting estimating equations, than by using the standard $\mathbf{A}$ matrix calculated with pedigree information solely.

## Conclusions

The ancestral regression (AR) and ancestral regression to parents (PAR) are causal Markov models that guarantee a positive dependence between breeding values of relatives which, in turn, result in sparse inverses of the respective covariance matrices. We have derived the covariances between the breeding values of relatives that are needed to fit the AR and PAR models to phenotypes, pedigree, and genotypes to obtain BLUP of breeding values. The inbreeding coefficient was also derived under gametic disequilibrium due to differential recombination of grandparental genomes in the gametes.

### Supplementary Information

**Additional file 1.** Lineages, pedigree, graphs, and breeding values [49–55]. **Figure S1.** Example of a DAG for a small pedigree. **Figure S2.** Total, direct, and indirect effects of breeding values in an ancestral regression model.

**Additional file 2.** Breeding values under gametic equilibrium and disequilibrium [56–65]. **Figure S3.** DAG representation of ancestral regression (AR). **Figure S4.** Acyclic mixed graph representation of ancestral regression to parents (PAR).

**Additional file 3.** Covariance matrix of breeding values under identity disequilibrium [66–68].

## Figures and Tables

**Fig. 1:**
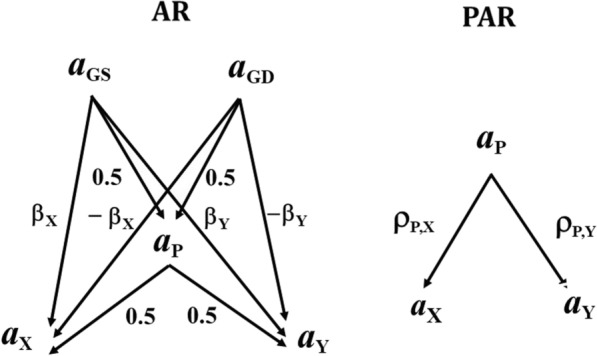
The AR and the PAR models for the breeding values of two half-sibs (X and Y), the common parent (P) and the parents of P (GS and GD)

## Data Availability

Not applicable.

## Variance of breeding values under the ancestral regression model (AR) and under the ancestral regression to parents (PAR)

By using the variance operator in the ancestral regression of Eq. (8) we obtain:

$$\text{Var}\left({a}_{\text{X}}\right)=\text{Var}(0.5{a}_{\text{S}}+0.5{a}_{\text{D}}+{\upbeta}_{\text{S}}\left({a}_{\text{SS}}-{a}_{\text{DS}}\right)+{\upbeta}_{\text{D}}\left({a}_{\text{SD}}-{a}_{\text{DD}}\right)+{\upphi}_{\text{X}})$$

$$\begin{array}{ll} =\text{Var}\left(0.5{a}_{\text{S}}+0.5{a}_{\text{D}}\right)+\text{Var}\left({\upbeta}_{\text{S}}\left({a}_{\text{SS}}-{a}_{\text{DS}}\right)+{\upbeta}_{\text{D}}\left({a}_{\text{SD}}-{a}_{\text{DD}}\right)\right) \\ \quad +\,2\text{cov}(0.5{a}_{\text{S}}+0.5{a}_{\text{D}}, {\upbeta}_{\text{S}}\left({a}_{\text{SS}}-{a}_{\text{DS}}\right)+{\upbeta}_{\text{D}}\left({a}_{\text{SD}}-{a}_{\text{DD}}\right)+\text{Var}({\upphi}_{\text{X}}) \end{array}$$

$$\begin{array}{ll}=0.25 \text{Var}\left({a}_{\text{S}}+{a}_{\text{D}}\right)+{\upbeta}_{\text{S}}^{2}\text{Var}\left({a}_{\text{SS}}-{a}_{\text{DS}}\right)+{\upbeta}_{\text{D}}^{2}\text{Var}\left({a}_{\text{SD}}-{a}_{\text{DD}}\right) \\ \quad +\,{2\upbeta}_{\text{S}}{\upbeta}_{\text{D}}\text{cov}\left({a}_{\text{SS}}-{a}_{\text{DS}}, {a}_{\text{SD}}-{a}_{\text{DD}}\right)\\ \quad + \,0.5 \text{cov}\left({a}_{\text{S}}+{a}_{\text{D}}, {\upbeta}_{\text{S}}\left({a}_{\text{SS}}-{a}_{\text{DS}}\right)\right)+0.5 \text{cov}({a}_{\text{S}}+{a}_{\text{D}}, {\upbeta}_{\text{D}}\left({a}_{\text{SD}}-{a}_{\text{DD}}\right)+\text{Var}({\upphi}_{\text{X}}) \end{array}$$

$$\begin{array}{ll}=0.25\left[\text{Var}\left({a}_{\text{S}}\right)+\text{Var}\left({a}_{\text{D}}\right)+2\text{cov}\left({a}_{\text{S}},{a}_{\text{D}}\right)\right]\\ \quad +\, {\upbeta}_{\text{S}}^{2}\left[\text{Var}\left({a}_{\text{SS}}\right)+\text{Var}\left({a}_{\text{DS}}\right)-2\text{cov}\left({a}_{\text{SS}},{a}_{\text{DS}}\right)\right] \\ \quad +\, {\upbeta}_{\text{D}}^{2}\left[\text{Var}\left({a}_{\text{SD}}\right)+\text{Var}\left({a}_{\text{DD}}\right)-2\text{cov}\left({a}_{\text{SD}},{a}_{\text{DD}}\right)\right]\\ \quad +\, {2\upbeta}_{\text{S}}{\upbeta}_{\text{D}}\text{cov}\left({a}_{\text{SS}},{a}_{\text{DS}}\right)-\text{cov}\left({a}_{\text{SS}},{a}_{\text{DD}}\right)-\text{cov}\left({a}_{\text{DS}},{a}_{\text{SD}}\right)+\text{cov}({a}_{\text{DS}},{a}_{\text{DD}}) \\ \quad +0.5{\upbeta}_{\text{S}}\left[\text{cov}\left({a}_{\text{S}},{a}_{\text{SS}}\right)-\text{cov}\left({a}_{\text{S}},{a}_{\text{DS}}\right)+\text{cov}\left({a}_{\text{D}},{a}_{\text{SS}}\right)-\text{cov}({a}_{\text{D}},{a}_{\text{DS}})\right] \\ \quad +\, 0.5\, {\upbeta}_{\text{D}}\left[\text{cov}\left({a}_{\text{S}},{a}_{\text{SD}}\right)-\text{cov}\left({a}_{\text{S}},{a}_{\text{DD}}\right)+\text{cov}\left({a}_{\text{D}},{a}_{\text{SD}}\right)-\text{cov}({a}_{\text{D}},{a}_{\text{DD}})\right]+\text{Var}({\upphi}_{\text{X}}), \end{array}$$

Finally, writing all the terms as a function of elements of ${\mathbf{\Sigma}}$ results in the following expression:

45 $$\text{Var}\left({a}_{\text{X}}\right)=\left\{0.25\left[2+{F}_{\text{S}}+{F}_{\text{D}}+2{\Sigma}_{\text{S},\text{D}}\right]+{\upbeta}_{\text{S}}^{2}\left[2+{F}_{\text{SS}}+{F}_{\text{DS}}-2{\Sigma}_{\text{SS},\text{DS}}\right]+{\upbeta}_{\text{D}}^{2}\left[2+{F}_{\text{SD}}+{F}_{\text{DD}}-2{\Sigma}_{\text{SD},\text{DD}}\right]+{2\upbeta}_{\text{S}}{\upbeta}_{\text{D}}\left[{\Sigma}_{\text{SS},\text{SD}}-{\Sigma}_{\text{SS},\text{DD}}-{\Sigma}_{\text{DS},\text{SD}}+{\Sigma}_{\text{DS},\text{DD}}\right]+0.5{\upbeta}_{\text{S}}\left[{\Sigma}_{\text{SS},\text{S}}-{\Sigma}_{\text{DS},\text{S}}+{\Sigma}_{\text{SS},\text{D}}-{\Sigma}_{\text{DS},\text{D}}\right]+0.5{\upbeta}_{\text{D}}\left[{\Sigma}_{\text{SD},\text{S}}-{\Sigma}_{\text{DD},\text{S}}+{\Sigma}_{\text{SD},\text{D}}-{\Sigma}_{\text{DD},\text{D}}\right]\right\}{\upsigma}_{\text{A}}^{2}+\text{Var}({\upphi}_{\text{X}}).$$

Application of the variance operator for the ancestral regression to parents (PAR) model, i.e., Eq. (25) results in being equal to:

46 $$\begin{array}{*{20}l} {{\text{Var}}_{{{\uprho}}} \left( {a_{{\text{X}}}} \right) = {\text{Var}}_{{{\uprho}}} \left( {{{\uprho}}_{{\text{S}}} a_{{\text{S}}} + {{\uprho}}_{{\text{D}}} a_{{\text{D}}}} \right) + {\text{Var}}_{{{\uprho}}} \left( {{{\upphi}}_{i}} \right)} \hfill \\ {\quad = {\uprho}_{{\text{S}}}^{2} {\text{Var}}_{{{\uprho}}} \left( {a_{{\text{S}}}} \right) + {{\uprho}}_{{\text{D}}}^{2} {\text{Var}}_{{{\uprho}}} \left( {a_{{\text{D}}}} \right) + 2{{\uprho}}_{{\text{S}}} {{\uprho}}_{{\text{D}}} \;{\text{cov}}_{\uprho} \left( {a_{{\text{S}}} ,a_{{\text{D}}}} \right) + {\text{Var}}_{{{\uprho}}} \left( {{{\upphi}}_{i}} \right)} \hfill \\ {\quad = \left[ {{{\uprho}}_{{\text{S}}}^{2} \left( {1 + F_{{\text{S}}}} \right) + {{\uprho}}_{{\text{D}}}^{2} \left( {1 + F_{{\text{D}}}} \right) + 2{{\uprho}}_{{\text{S}}} {{\uprho}}_{{\text{D}}} \;{\Sigma} _{{{{\uprho S}},{\text{D}}}}} \right] {{\Sigma}}_{{\text{A}}}^{2} + {\text{Var}}\left( {{{\upphi}}_{i}} \right),} \hfill \\ \end{array}$$

## Inbreeding under the models of ancestral regression and the ancestral regression to parents

Wright [28] “defined” the coefficient of inbreeding of individual $\text{X}$ (${F}_{(\text{W})}$) as ${F}_{\text{X}(\text{W})}=0.5{r}_{\text{SD}}\sqrt{\left(1+{F}_{\text{S}(\text{W})}\right)\left(1+{F}_{\text{D}(\text{W})}\right)}$, where ${F}_{\text{S}(\text{W})}$ and ${F}_{\text{D}(\text{W})}$ are the inbreeding of the sire and dam of $\text{X}$, respectively, and ${r}_{\text{SD}}$ is the correlation between the parental breeding values ${r}_{\text{SD}}=\text{cov}({a}_{\text{S}},{a}_{\text{D}})/\sqrt{\text{Var}\left({a}_{\text{S}}\right)\text{Var}({a}_{\text{D}})}$. By replacing with this correlation in ${F}_{\text{X}(\text{W})}$, we obtain the value of the inbreeding of $\text{X}$ under the AR model (${F}_{\text{X}}$) in the following manner:

47 $$\begin{array}{ll}{F}_{\text{X}} &=0.5\left[\frac{\text{cov}({a}_{\text{S}},{a}_{\text{D}})}{\sqrt{\text{Var}\left({a}_{\text{S}}\right)\text{Var}({a}_{\text{D}})}}\right]\sqrt{\left(1+{F}_{\text{S}}\right)\left(1+{F}_{\text{D}}\right)} \\ & =0.5\text{cov}\left({a}_{\text{S}},{a}_{\text{D}}\right) \\ & =0.5\text{cov}\left({a}_{\text{S}}+{\upbeta}_{\text{S}}\left({a}_{\text{SS}}-{a}_{\text{DS}}\right), {a}_{\text{D}}+{\upbeta}_{\text{D}}\left({a}_{\text{SD}}-{a}_{\text{DD}}\right)\right) \\ & =0.5\left[\text{cov}\left({a}_{\text{S}},{a}_{\text{D}}\right)+\text{cov}\left({a}_{\text{S}},{\upbeta}_{\text{D}}\left({a}_{\text{SD}}-{a}_{\text{DD}}\right)\right)+\text{cov}\left({\upbeta}_{\text{S}}\left({a}_{\text{SS}}-{a}_{\text{DS}}\right), {a}_{\text{D}}\right)+\text{cov}({\upbeta}_{\text{S}}\left({a}_{\text{SS}}-{a}_{\text{DS}}\right), {\upbeta}_{\text{D}}\left({a}_{\text{SD}}-{a}_{\text{DD}}\right))\right] \\ & =0.5\left[{\Sigma}_{\text{S},\text{D}}+{\upbeta}_{\text{D}}\left({\Sigma}_{\text{SD},\text{S}}-{\Sigma}_{\text{DD},\text{S}}\right)+{\upbeta}_{\text{S}}\left({\Sigma}_{\text{SS},\text{D}}-{\Sigma}_{\text{DS},\text{D}}\right)\right] \\ & \ \quad +\,\text{cov}({\upbeta}_{\text{S}}\left({a}_{\text{SS}}-{a}_{\text{DS}}\right),{\upbeta}_{\text{D}}\left({a}_{\text{SD}}-{a}_{\text{DD}}\right)). \end{array}$$

Consider now the cross-covariance between the paternal and maternal identity disequilibrium terms:

48 $$\begin{array}{ll}\text{cov}\left({\upbeta}_{\text{S}}\left({a}_{\text{SS}}-{a}_{\text{DS}}\right),{\upbeta}_{\text{D}}\left({a}_{\text{SD}}-{a}_{\text{DD}}\right)\right)={\upbeta}_{\text{S}}{\upbeta}_{\text{D}}\text{cov}\left({a}_{\text{SS}}-{a}_{\text{DS}}, {a}_{\text{SD}}-{a}_{\text{DD}}\right) \\ \quad =\, {\upbeta}_{\text{S}}{\upbeta}_{\text{D}}\left[\text{cov}\left({a}_{\text{SS}}, {a}_{\text{DS}}\right)-\text{cov}\left({a}_{\text{SS}}, {a}_{\text{DD}}\right)-\text{cov}\left({a}_{\text{DS}}, {a}_{\text{SD}}\right)+\text{cov}({a}_{\text{DS}}, {a}_{\text{DD}})\right] \\ \quad =\,{\upbeta}_{\text{S}}{\upbeta}_{\text{D}}\left[{\Sigma}_{\text{SS},\text{SD}}-{\Sigma}_{\text{SS},\text{DD}}-{\Sigma}_{\text{DS},\text{SD}}+{\Sigma}_{\text{DS},\text{DD}}\right]. \end{array}$$

If we replace with Eq. (48) in Eq. (47), inbreeding under AR is equal to:

49 $${F}_{\text{X}}=0.5\left[{\Sigma}_{\text{SD}}+{\upbeta}_{\text{S}}\left({\Sigma}_{\text{SS},\text{D}}-{\Sigma}_{\text{DS},\text{D}}\right)+{\upbeta}_{\text{D}}\left({\Sigma}_{\text{SD},\text{S}}-{\Sigma}_{\text{DD},\text{S}}\right)+{\upbeta}_{\text{S}}{\upbeta}_{\text{D}} ({\Sigma}_{\text{SS},\text{SD}}-{\Sigma}_{\text{SS},\text{DD}}-{\Sigma}_{\text{DS},\text{SD}}+{\Sigma}_{\text{DS},\text{DD}})\right].$$

For the PAR model inbreeding is defined to be equal to:

50 $$F_{\rho_{\text {X}}}= 2 {\uprho}_{\text{S}} {\uprho}_{\text{D}} {\Sigma}_{{\rho_{\text{S}}},\text{D}}.$$

Hence, $${\text{Var}}_{\uprho}\left({a}_{\text{X}}\right)$$ is:

51 $${\text{Var}}_{\uprho}\left({a}_{\text{X}}\right)=\left(1+ {F}_{{\rho {\rm {X}}}}\right){\upsigma}_{\text{A}}^{2},$$

such that $${\uprho}_{\text{S}}^{2} \left(1+{F}_{{\uprho {\text{S}}}}\right)+{\uprho}_{\text{D}}^{2} \left(1+{F}_{{\rho {\text{D}}}}\right)+{\text{Var}}_{\uprho}\left({\upphi}_{\text{i}}\right){\left({\upsigma}_{\text{A}}^{2}\right)}^{-1}$$ = 1.

## Covariance between breeding values under the ancestral regression model when neither animal is an ancestor of each other

Let the BV of the related individuals $\text{X}$ and $\text{Y}$ be ${a}_{\text{X}}$ and ${a}_{\text{Y}}$. The notation, for example, will be that $\text{DDY}$ is the maternal granddam of $\text{Y}$, $\text{SSX}$ is the paternal grandsire of $\text{X}$, $\text{SX}$ is the sire of $\text{X}$ and $\text{DY}$ the dam of $\text{Y}$. By using the covariance operator in the AR model when neither individual is an ancestor of each other, the covariance between the BV of $\text{X}$ and $\text{Y}$ is equal to:

$$\begin{array}{ll}{\Sigma}_{\text{X},\text{Y}}=\text{cov}\left[0.5\left({a}_{\text{SX}}+{a}_{\text{DX}}\right)+{\upbeta}_{\text{SX}}\left({a}_{\text{SSX}}-{a}_{\text{DSX}}\right)+{\upbeta}_{\text{DX}}\left({a}_{\text{SDX}}-{a}_{\text{DDX}}\right), 0.5\left({a}_{\text{SY}}+{a}_{\text{DY}}\right)+{\upbeta}_{\text{SY}}\left({a}_{\text{SSY}}-{a}_{\text{DSY}}\right)+{\upbeta}_{\text{DY}}\left({a}_{\text{SDY}}-{a}_{\text{DDY}}\right)\right] \\ =0.25\text{cov}\left[{a}_{\text{SX}}+{a}_{\text{DX}},{a}_{\text{SY}}+{a}_{\text{DY}}\right] \\ \quad +\,0.5{\upbeta}_{\text{SY}}\text{cov}\left[{a}_{\text{SX}}+{a}_{\text{DX}},{a}_{\text{SSY}}-{a}_{\text{DSY}}\right]\\ \quad +\,0.5{\upbeta}_{\text{DY}}\text{cov}\left[{a}_{\text{SX}}+{a}_{\text{DX}}, {a}_{\text{SDY}}-{a}_{\text{DDY}}\right]\\ \quad +\,0.5{\upbeta}_{\text{SX}}\text{cov}\left[{a}_{\text{SSX}}-{a}_{\text{DSX}}, {a}_{\text{SY}}+{a}_{\text{DY}}\right]\\ \quad +\,{\upbeta}_{\text{SX}}{\upbeta}_{\text{SY}}\text{cov}\left[{a}_{\text{SSX}}-{a}_{\text{DSX}},{a}_{\text{SSY}}-{a}_{\text{DSY}}\right]\\ \quad +\,{\upbeta}_{\text{SX}}{\upbeta}_{\text{DY}}\text{cov}\left[{a}_{\text{SSX}}-{a}_{\text{DSX}},{a}_{\text{SDY}}-{a}_{\text{DDY}}\right]\\ \quad +\,0.5{\upbeta}_{\text{DX}}\text{cov}\left[{a}_{\text{SDX}}-{a}_{\text{DDX}}, {a}_{\text{SY}}+{a}_{\text{DY}}\right]\\ \quad +\,{\upbeta}_{\text{DX}}{\upbeta}_{\text{SY}}\text{cov}\left[{a}_{\text{SDX}}-{a}_{\text{DDX}},{a}_{\text{SSY}}-{a}_{\text{DSY}}\right]\\ \quad +\,{\upbeta}_{\text{DX}}{\upbeta}_{\text{DY}}\text{cov}\left[{a}_{\text{SDX}}-{a}_{\text{DDX}},{a}_{\text{SDY}}-{a}_{\text{DDY}}\right], \end{array}$$

$$\begin{array}{ll}=0.25\left[{\Sigma}_{\text{SX},\text{SY}}+{\Sigma}_{\text{SX},\text{DY}}+{\Sigma}_{\text{DX},\text{SY}}+{\Sigma}_{\text{DX},\text{DY}}\right] \\ \quad +\,0.5{\upbeta}_{\text{SY}}\left[{\Sigma}_{\text{SX},\text{SSY}}-{\Sigma}_{\text{SX},\text{DSY}}+{\Sigma}_{\text{DX},\text{SSY}}-{\Sigma}_{\text{DX},\text{DSY}}\right]\\ \quad +\,0.5{\upbeta}_{\text{DY}}\left[{\Sigma}_{\text{SX},\text{SDY}}-{\Sigma}_{\text{SX},\text{DDY}}+{\Sigma}_{\text{DX},\text{SDY}}-{\Sigma}_{\text{DX},\text{DDY}}\right]\\ \quad +\,0.5{\upbeta}_{\text{SX}}\left[{\Sigma}_{\text{SSX},\text{SY}}+{\Sigma}_{\text{SSX},\text{DY}}-{\Sigma}_{\text{DSX},\text{SY}}-{\Sigma}_{\text{DSX},\text{DY}}\right]\\ \quad +\,0.5{\upbeta}_{\text{DX}}\left[{\Sigma}_{\text{SDX},\text{SY}}+{\Sigma}_{\text{SDX},\text{DY}}-{\Sigma}_{\text{DDX},\text{SY}}-{\Sigma}_{\text{DDX},\text{DY}}\right]\\ \quad +\,{\upbeta}_{\text{SX}}{\upbeta}_{\text{SY}}\left[{\Sigma}_{\text{SSX},\text{SSY}}-{\Sigma}_{\text{SSX},\text{DSY}}-{\Sigma}_{\text{DSX},\text{SSY}}+{\Sigma}_{\text{DSX},\text{DSY}}\right]\\ \quad +\,{\upbeta}_{\text{SX}}{\upbeta}_{\text{DY}}\left[{\Sigma}_{\text{SSX},\text{SDY}}-{\Sigma}_{\text{SSX},\text{DDY}}-{\Sigma}_{\text{DSX},\text{SDY}}+{\Sigma}_{\text{DSX},\text{DDY}}\right]\\ \quad +\,{\upbeta}_{\text{DX}}{\upbeta}_{\text{SY}}\left[{\Sigma}_{\text{SDX},\text{SSY}}-{\Sigma}_{\text{SDX},\text{DSY}}-{\Sigma}_{\text{DDX},\text{SSY}}+{\Sigma}_{\text{DDX},\text{DSY}}\right]\\ \quad +\,{\upbeta}_{\text{DX}}{\upbeta}_{\text{DY}}\left[{\Sigma}_{\text{SDX},\text{SDY}}-{\Sigma}_{\text{SDX},\text{DDY}}-{\Sigma}_{\text{DDX},\text{SDY}}+{\Sigma}_{\text{DDX},\text{DDY}}\right], \end{array}$$

The final expression is equal to:

52 $${\Sigma}_{\text{X},\text{Y}}=0.5\left\{0.5\left[{\Sigma}_{\text{SX},\text{SY}}+{\Sigma}_{\text{SX},\text{DY}}+{\Sigma}_{\text{DX},\text{SY}}+{\Sigma}_{\text{DX},\text{DY}}\right]+{\upbeta}_{\text{SY}}\left[{\Sigma}_{\text{SX},\text{SSY}}-{\Sigma}_{\text{SX},\text{DSY}}+{\Sigma}_{\text{DX},\text{SSY}}-{\Sigma}_{\text{DX},\text{DSY}}\right]+{\upbeta}_{\text{DY}}\left[{\Sigma}_{\text{SX},\text{SDY}}-{\Sigma}_{\text{SX},\text{DDY}}+{\Sigma}_{\text{DX},\text{SDY}}-{\Sigma}_{\text{DX},\text{DDY}}\right]+{\upbeta}_{\text{SX}}\left[{\Sigma}_{\text{SSX},\text{SY}}+{\Sigma}_{\text{SSX},\text{DY}}-{\Sigma}_{\text{DSX},\text{SY}}-{\Sigma}_{\text{DSX},\text{DY}}\right]+{\upbeta}_{\text{DX}}\left[{\Sigma}_{\text{SDX},\text{SY}}+{\Sigma}_{\text{SDX},\text{DY}}-{\Sigma}_{\text{DDX},\text{SY}}-{\Sigma}_{\text{DDX},\text{DY}}\right]\right\}+{\upbeta}_{\text{SX}}\left\{{\upbeta}_{\text{SY}}\left[{\Sigma}_{\text{SSX},\text{SSY}}-{\Sigma}_{\text{SSX},\text{DSY}}-{\Sigma}_{\text{DSX},\text{SSY}}+{\Sigma}_{\text{DSX},\text{DSY}}\right]+{\upbeta}_{\text{DY}}\left[{\Sigma}_{\text{SSX},\text{SDY}}-{\Sigma}_{\text{SSX},\text{DDY}}-{\Sigma}_{\text{DSX},\text{SDY}}+{\Sigma}_{\text{DSX},\text{DDY}}\right]\right\}+{\upbeta}_{\text{DX}}\left\{{\upbeta}_{\text{SY}}\left[{\Sigma}_{\text{SDX},\text{SSY}}-{\Sigma}_{\text{SDX},\text{DSY}}-{\Sigma}_{\text{DDX},\text{SSY}}+{\Sigma}_{\text{DDX},\text{DSY}}\right]+{\upbeta}_{\text{DY}}\left[{\Sigma}_{\text{SDX},\text{SDY}}-{\Sigma}_{\text{SDX},\text{DDY}}-{\Sigma}_{\text{DDX},\text{SDY}}+{\Sigma}_{\text{DDX},\text{DDY}}\right]\right\}.$$

This is a formidable expression but will be generally much less involved because many of the elements of ${\varvec{\Sigma}}$ will be zero, especially if inbreeding is absent.

As an example, consider the covariances among full-sibs and half-sibs in the absence of inbreeding. For full-sibs ${\text{S}}_{\text{X}}={\text{S}}_{\text{Y}}$, ${\text{D}}_{\text{X}}={\text{D}}_{\text{Y}}$, ${\text{SS}}_{\text{X}}={\text{SS}}_{\text{Y}}$, ${\text{DS}}_{\text{X}}={\text{DS}}_{\text{Y}}$, ${\text{SD}}_{\text{X}}={\text{S}}_{\text{DY}}$, and ${\text{DD}}_{\text{X}}={\text{DD}}_{\text{Y}}$. Then, order the individuals $\text{SS}$, $\text{DS}$, $\text{SD}$, $\text{DD}$, $\text{S}$, and $\text{D}$ in a matrix array that contains the relationships for the ancestors of $\text{X}$ (in the rows) with the ancestors of $\text{Y}$ (in the columns), which results in:

$${\Sigma}_{FS}=0.5+2 \left({\upbeta}_{\text{SX}}{\upbeta}_{\text{SY}} +{\upbeta}_{\text{DX}}{\upbeta}_{\text{DY}}\right)$$

Using these elements in Eq. ( 52) results in:

53 $${\Sigma}_{FS}=0.5+2 \left({\upbeta}_{\text{SX}}{\upbeta}_{\text{SY}} +{\upbeta}_{\text{DX}}{\upbeta}_{\text{DY}}\right)$$

By a similar reasoning, we obtain the following expressions for the relationships between paternal and maternal half-sibs, respectively:

$${\Sigma}_{PHS}=0.25+2{\upbeta}_{\text{SX}}{\upbeta}_{\text{SY}}$$

54 $${\Sigma}_{MHS}=0.25+2{\upbeta}_{\text{DX}}{\upbeta}_{\text{DY}}$$

It can be inferred that twice the product of the β parameters of the two sibs from either the paternal or maternal (half-sibs), or for both (full-sibs), results in the value of identity disequilibrium. Also, please note that the magnitude of a half-sib relationship can be greater than 0.25 whenever $\text{X}$ and $\text{Y}$ possess $\upbeta$ with the same sign, meaning that both half-sibs are more alike the same grandparent. Conversely, when $\text{X}$ and $\text{Y}$ differ in the signs of their $\upbeta$, the covariance between BV is smaller than 0.25. The resemblance is less clear for full-sibs because their $\upbeta$ need to agree in sign *simultaneously* with respect to the paternal and maternal sources of resemblance.

## The AR and PAR models are not equivalent

Two causal models are “Markov equivalent” whenever their associated direct acyclic graph (DAG) captures the same dependence–independence structure and the same set of conditional independencies [47, 48]. As an example, Appendix Fig. 1 displays the DAG of half-sibs in the absence of any inbreeding of the grandparents ($\text{GS}$ and $\text{GD}$), the common parent ($\text{P}$) and both half-sibs, for the AR and the PAR models. Please note that there are extra treks in the AR model going from ${a}_{\text{X}}$ to ${a}_{\text{Y}}$ through each grandparent without passing by the BV of the parent.

Reading out the DAG in Appendix Fig. 1, the covariance between the BV of $\text{X}$ and $\text{Y}$ involves two parameters in both the AR (${\upbeta}_{\text{X}}$ and ${\upbeta}_{\text{Y}}$) and PAR (${\uprho}_{\text{P},\text{X}}$ and ${\uprho}_{\text{P},\text{Y}}$) models. However, in the PAR model ${a}_{\text{X}}$ and ${a}_{\text{Y}}$ are conditionally independent on the BV of their grandparents $\text{GS}$ and $\text{GD}$ given that of their common parent $\text{P}$, thus the edges are not represented in the DAG. As a result, ${a}_{\text{X}}$ is conditionally independent of ${a}_{\text{Y}}$ given ${a}_{\text{P}}$, ${a}_{\text{GS}}$ and ${a}_{\text{GD}}$ under the AR model, while under the PAR model ${a}_{\text{X}}$ is conditionally independent of ${a}_{\text{Y}}$ given ${a}_{\text{P}}$ only.

Please note that, in Appendix Fig. 1, the regression coefficients ${\upbeta}_{\text{X}}$, $-{\upbeta}_{\text{X}}$, ${\upbeta}_{\text{Y}}$ and ${-\upbeta}_{\text{Y}}$ directly connect ${a}_{\text{GS}}$ and ${a}_{\text{GD}}$ to ${a}_{\text{X}}$ and ${a}_{\text{Y}}$. These path (or regression) coefficients that are worth plus or minus beta are called *chords* and the graph that contains them is named a *chordal graph* [46], whereas the graphs that do not contain chords are called *chordless graphs*. Consequently, AR is a chordal graph whereas PAR is a chordless graph. Theorem 2 in [48] says that two graphs are Markov equivalent models if and only if they do not contain any chordless *collider* structure in four nodes. A collider dependence structure (see page 10 in [4]) is that for “progeny in common”:

$${a}_{\text{GP}1}-0.5\to {a}_{\text{Sire}}-0.5\to \text{X}\leftarrow 0.5-{a}_{\text{Dam}}\leftarrow 0.5-{a}_{\text{GP}2}$$

or for “grand-progeny in common”:

$${a}_{\text{GS}}-{\upbeta}_{\text{X}},\to \text{X}\leftarrow -{\upbeta}_{\text{X}}-{a}_{\text{GD}} \, {\rm and}\, {a}_{\text{GS}}-{\upbeta}_{\text{Y}},\to \text{Y}\leftarrow -{\upbeta}_{\text{Y}}-{a}_{\text{GD}},$$

in the same DAG. Colliders can occur in chordal and chordless graphs, whereas non-collider structures such as the *lineage* (1 → 2 → 3 → 4) or the “common ancestor” (CA in 4 ← 2 ← CA → 1 → 3) can only occur in chordless graphs. Hence, for pedigrees with four or more animals, the chordless PAR has a different conditional dependence structure than the chordal AR, and thus they are not Markov equivalent models.

Also, the AR and PAR models are not equivalent in the sense of Henderson [36], who defined two models as equivalent when they have the same covariance matrix. We will show this by a numerical example. Let ${\upbeta}_{\text{S}}$ = 0.1166 and ${\upbeta}_{\text{D}}$ = 0.1226, and the matrix in Eq. (17) be equal to:

$${{\mathbf{\Sigma}}}_{\text{A}}=\left[\begin{array}{cccccc}1& 0& 0& 0& 0.5& 0\\ 0& 1& 0& 0& 0& 0\\ 0& 0& 1& 0& 0& 0.5\\ 0& 0& 0& 1& 0& 0.5\\ 0.5& 0.5& 0& 0& 1& 0\\ 0& 0& 0.5& 0.5& 0& 1\end{array}\right]$$

Use of Eq. (30) results in the covariance between the sire and $\text{X}$ under the AR model being equal to:

$${\Sigma}_{\text{S},\text{X}}=0.5\left(1.0148+0\right)+0.1166\left(0.50-0.5365\right)+0.1226\left(0-0.0443\right)=0.5085$$

In turn, we can derive the covariance between the BV of the sire and $\text{X}$ under the PAR model as follows:

$${\text{cov}}_{\uprho}\left({a}_{\text{S}}, {a}_{\text{X}}\right)={\text{cov}}_{\uprho}({a}_{\text{S}}, {\uprho}_{\text{S}}, {a}_{\text{S}}+{\uprho}_{\text{D}}{a}_{\text{D}})={\text{cov}}_{\uprho}({a}_{\text{S}}, {\uprho}_{\text{S}}, {a}_{\text{S}})+{{\text{cov}}_{\uprho}({a}_{\text{S}},\uprho}_{\text{D}}{a}_{\text{D}})={\uprho}_{\text{S}}\left(1+{F}_{{\rho {\rm S}}}\right)+{\uprho}_{\text{D}}{\Sigma}_{{\rho {\rm DS}}}$$

Now, to calculate the numerical value of this covariance, we need to know the values of the $\uprho$ coefficients. These are obtained by solving the system of Eq. (23) or Eq. (24). In this example:

$$\left[\begin{array}{cc}0.4917& -0.0088\\ -0.0088& 0.4864\end{array}\right]\left[\begin{array}{c}{\uprho}_{\text{S}}\\ {\uprho}_{\text{D}}\end{array}\right]=\left[\begin{array}{c}0.2547\\ 0.2478\end{array}\right]$$

with solution ${\uprho}_{\text{S}}$ = 0.528 and ${\uprho}_{\text{D}}$ = 0.519. Then, the covariance is equal to:

$${\text{cov}}_{\text{PAR}}\left({a}_{\text{S}}, {a}_{\text{X}}\right)=0.5274\left(1.0148\right)+0.519\left(0\right)=0.5352$$

Since 0.5352 ≠ 0.5085, the AR and PAR models are not covariance equivalent in the sense of [36].
