# Supplementary material for: Causal inference for the covariance between breeding values under identity disequilibrium
Source: Genet Sel Evol. 2022 Sep 23;54:64. doi: 10.1186/s12711-022-00750-6 (PMC9502921; doi:10.1186/s12711-022-00750-6)
Supplement: Supplementary file 1 — Additional file 1. Lineages, pedigree, graphs, and breeding values [49–55]. Figure S1. Example of a DAG for a small pedigree. Figure S2. Total, direct, and indirect effects of breeding values in an ancestral regression model. [file 12711_2022_750_MOESM1_ESM.docx]

**Additional file 1**

**Lineages, pedigree, graphs, and breeding values**

Because the parental genomes that are passed to the offspring in the gametes are the results of independent meiosis, Li [49] (see page 172) equated the genotypic effect of an individual to the sum of the maternal (*g*_D_) and paternal gametic effects (*g*_S_). Starting from the “founder” animal (e.g., 0) to the youngest one (animal *K*), a *lineage* is a set of adjacent gametic effects ordered such that *g*_0_ → *g*_1_ → *g*_2 …_ → *g_K_*, with the arrows indicating that animal *k* is the sire or the dam of individual *k*+1. The lineages represent the genome flow through uniparental *effects* from the gametes, instead of biparental breeding values (BV). Each arrow has a value equal to a *standardized partial regression coefficient* or *path coefficient* (Wright [27]). The *single-*headed arrow has a *causal* meaning [4, 49]. The association of BV to causal effects is a stochastic statement (see page 362 in Arjas and Eerola [50],) that indicates that the probability of the BV of an individual, given that the animal has received from each of its parent a specific portion of their genome, is strictly greater than the *marginal probability* of the BV of the offspring: *P* (BV_Offspring_ | BV_Parent_) − *P* (BV_Offspring_) > 0. This is tantamount to saying that parental BV are *causal* whenever the parents add enough information on the BV of their offspring, such that the conditional probability of its BV given those of its parents is greater than the marginal probability of the BV of their offspring.

A pedigree is a collection of intersecting lineages and the random variables involved are BV rather than gametic effects. If the pedigree is perfectly known, it can be represented by a *directed acyclic graph* (DAG, [4]). The DAG is *directed* and *acyclic* because the arrows always point in the direction of the youngest animal, such that the genomes never flow backwards to the ancestors. Wright [27, 40] also considered the *correlation* of the random variables in the graph, and he used the double-headed arrows (↔) as a symbol of correlation. When the graph includes single- and double-headed arrows, the stochastic representation is the *Ancestral Graph* [51] or, more generally, the *Acyclic Mixed Graph* (AMG, [39]). The AMG provides formal probabilistic statements and generalizes the notion of a *path coefficient diagram* by Wright [27, 40]. Since, in our paper, BV are standardized random variables, the AMG conveniently allows us to calculate inbreeding as the cov(*a*_S_, *a*_D_) by the *path* rule of Wright [40] that in its more recent version from the acyclic mixed graphs is the *trek rule* (see Lemma 3.1 in [39]). This rule indicates that the correlation (covariance) between two breeding values results from the multiplication of the values of all path coefficients in the AMG from one random variable to the other but traversing only one correlation. Thus, in the trek rule only one *change of direction* is allowed [39, 40, 49], and can be observed in the following scheme:

*a*_X_ ← *a*_X−1_ ← … ← *a*_X1_ ← *a*_CA_ → *a*_Y1_ → … → *a*_Y−1_ → *a*_Y_.

The numerical subscripts prior to the minus signs indicate the previous generations from X and Y to the common ancestor (CA): 1, 2, and so on up to X1 and Y1, which are the progeny of CA. As an example, consider the set of BV from the three-generation pedigree in Fig.1. It includes two lineages starting from grandparent A, then its progeny B, and two grand-progeny (X and Y).

**Additional file 1 Figure S1:** **Example of a DAG for a small pedigree**


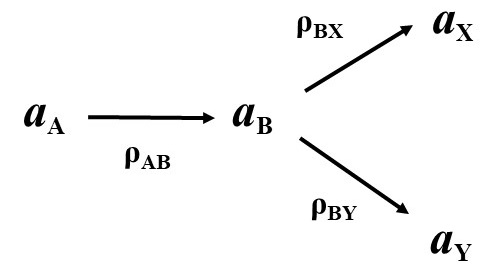


Using Wright’s rule, the cov(*a*_A_, *a*_Y_) is equal to the product of ρ_AB_ and ρ_BY_ because the only path from A to Y is through B. Please note that in Additional file 1 Figure S1 there are no arrows pointing to the founder because its parents are not known. Also, the covariance between the BV of the half-sibs, cov(*a*_X_, *a*_Y_) is equal to the product of ρ_BX_ and ρ_BY_ and passes also through B.

In Additional file 1 Figure S1, the DAG for the BV in the lineage from A to X is $a_{A} \to a_{B} \to a_{X}$. The covariances between BV in this lineage is such that cov(*a*_A_, *a*_X_) < cov(*a*_B_, *a*_X_). In general, the inequality becomes greater as the number of generations separating the ancestor and X becomes larger, a fact that is seen in the first column or row of the $\mathbf{A}$ matrix of the following matrix of the DAG:

$$\mathbf{A=}\left[ \begin{matrix} 1 & 0.50 & 0.25 \\ 0.50 & 1 & 0.50 \\ 0.25 & 0.50 & 1 \end{matrix} \right] \mathbf{A}^{-1}\mathbf{=}\left[ \begin{matrix} 4/3 & -2/3 & 0 \\ -2/3 & 5/3 & -2/3 \\ 0 & 0.50 & 4/3 \end{matrix} \right]$$

Wermuth and Cox [52] noted that the inverse covariance matrix, **A**^−1^, displays the *partial correlations* among random variables after conditioning on the variables from their parents. These covariances are exactly equal to the covariance among residual breeding values, i.e., cov(*a*_A_, (*a*_X_|*a*_B_)) = 0 = $\mathbf{A}_{1,3}^{-1}$= cov(ϕ_A_, ϕ_X_). The reason is that in a Gaussian distribution, conditional correlations (covariances) and partial (residual) correlations (covariances) are equal (Baba et al [53]). Conditional independence arises because *a*_B_ is “blocking”, in the language of Pearl [4], the *flow of information* between *a*_A_ and *a*_X_, and the direction of the conditioning results in independent residual breeding values. Pearl [4] called this conditioning in which the resulting random variables become independent, *d*−*separation*. In the setting of breeding value prediction, the *d*-separation criterion reflects that, once *a*_B_ is given, *a*_A_ cannot add any new information to predict *a*_X_ which is not already in *a*_B_.

***Direct, indirect, and total effects on breeding values***

Pearl [4] discussed total, direct and indirect effects in causal inference. Additional file 1 Figure S2 displays a path coefficient diagram including the BV of an individual X, a parent P (either the dam or the sire), and those of both parents of P (SP and DP) under the ancestral regression model (AR).

**Additional file 1 Figure S2:** **Total, direct, and indirect effects of breeding values in an ancestral regression model.**


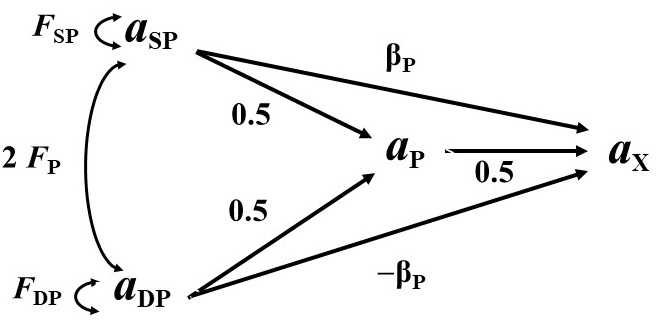


In AR, the *indirect effect* [4] of *a*_DP_ or *a*_SP_ on *a*_X_ results from the transmission of half the BV of GP (either DP or SP) to P, and then the transmission of half the BV of P to X. By using the tracing rules of path coefficients [27, 40], the value of the indirect effect of *a*_GP_ to *a*_X_ is 0.25 (= 0.5^2^). However, the *direct effect* (see section 4.5 in Pearl [4]) has a value of $\beta_{X,GPP}$ and goes directly [4] from *a*_GP_ to *a*_X._. These differential contributions from the BV of grandparents to the BV of the individual are not accounted for in the animal model [1] where they merge into the Mendelian residual$\phi_{X}$.

A theorem by Cochran [54] constraints the $\beta_{X,GPP}$ parameter such that the *total effect* [4] of *a*_GP_ to *a*_X_ is the sum of the direct plus the indirect effects, and it is equal to the *total effect* ($\beta_{X, \mathrm{GP}}$, [4]) of the BV of GP onto that of X, as follows:

$\beta_{X, \mathrm{GP}}= \beta_{X, GPP}+ \beta_{X, P\mathrm{GP}} \beta_{P, GP}= \beta_{X, GPP}+ \left( 0.5 \right)\left( 0.5 \right)=\beta_{X, GPP}+0.25.$ (S1.1)

This total effect is a *compounded path* in the terms of Wright [27], or a *triangular* graph in terms of an acyclic mixed graphs [39], because it is *decomposable* in the direct effect ($\beta_{X, \mathrm{GP}\left| P \right.}$) and the indirect effect (0.25). A similar expression to Eq. (S1.1) is obtained from the other grandparent of X. Adding both expressions and equating the result to the total contribution of each parent, which is equal to 0.5, results in:

$0.5= \beta_{X, SP}+ 0.25+\beta_{X, DP}+ 0.25.$ (S1.2)

As there is only one degree of freedom to estimate $\beta_{X,SPP}$and $\beta_{X,DPP}$, we need over-identification restrictions (see p 42-49 in Kenny [55] to end up with one parameter from each parental meiosis and the following path coefficients:

$\beta_{X,SSS}=-\beta_{X,DSS}= \beta_{S} \beta_{X,SDD}=-\beta_{X,DDD}= \beta_{D}$. (S1.3)

***Markov properties of the distribution of breeding values***

A property of the distribution of BV is the “ordered Markov Condition” (Theorem 1.2.6, see p 19 in Pearl [4]), which we may call the “gametic” Markov property for BV. A necessary and sufficient condition for *f*(***a***) to be gametic Markov is that, conditional on the BV of their parents, each BV is independent of *all its predecessors in a lineage*. Suppose that, in Additional file 1 Figure S1, a parent of A, let’s say G, is added to the lineage. Then, the gametic Markov property tells us that$a_{G}\perp a_{X} \left| a_{B} \right., a_{G}$, in addition to the already observed conditional independence $a_{A}\perp a_{X} \left| a_{B} \right.$. This latter statement is implied by the “parental Markov condition” (Theorem 1.2.7, see p 19 in [4]). Under the parental Markov property, each BV is independent of *all its non–descendants in a pedigree*, i.e., *collaterals*: sibs, uncles, cousins, among others, and *ancestors*) conditional on the BV of their parents. As a consequence of the gametic Markov property when calculating the covariance between and ancestor (*a*_G_) and a descendant (*a*_X_), backward expansion of *a*_G_ is not possible because the ancestors of G in the lineage are *d*–separated from *a*_X_, and the conditional covariance between BV in *f*(***a***) will be 0.
