# Supplementary material for: Causal inference for the covariance between breeding values under identity disequilibrium
Source: Genet Sel Evol. 2022 Sep 23;54:64. doi: 10.1186/s12711-022-00750-6 (PMC9502921; doi:10.1186/s12711-022-00750-6)
Supplement: Supplementary file 2 — Additional file 2. Breeding values under gametic equilibrium and disequilibrium [56–65]. Figure S3. DAG representation of ancestral regression (AR). Figure S4. Acyclic mixed graph representation of ancestral regression to parents (PAR). [file 12711_2022_750_MOESM2_ESM.docx]

**Additional file 2**

**Breeding values under gametic equilibrium and disequilibrium**

More than 30 years ago, Kennedy et al. [56] described several useful properties of “animal models” for permanent genetic evaluation. They concluded that: (1) “through” the additive relationship matrix **A**, breeding values are expressed as *linear functions* of ancestral breeding values (BV) plus a Mendelian error term; and (2) BV “can be expressed in terms of separate gametic contributions of each parent by gametic models”. The first comment suggests that Kennedy et al. [56] viewed BV as random variables that result from a recursive linear process to ancestral BV: a “regression” that was related to a pedigree-dependent matrix **A**. The second statement sets the recursion to the sum of random gametic effects from the independent meioses that form the constituting gametes of the newborn animal. In this section, we review this linear recursive process of BV formation under gametic equilibrium and disequilibrium and its relationship with the covariance matrix of BV.

***Breeding values under gametic equilibrium***

Schaeffer et al. [57] expressed the BV of animal X in gametic equilibrium (*a*_*X_) as a function of gametic effects that originated from the sire $\left( g_{S_{X}} \right)$ and dam $\left( g_{D_{X}} \right)$ of X, as follows:

$a_{*X} = g_{S_{X}} + g_{D_{X}}$ (S2.1)

The paternal and maternal gametic effects in Eq. (S2.1) are respectively equal to:

$g_{S_{X}} = 0.5 a_{*S} + \delta_{S} g_{D_{X}}= 0.5 a_{*D} + \delta_{D}$ (S2.2)

The terms δ_D_ and δ_S_ indicate the residual gametic effects for the dam and the sire, these effects being uncorrelated even if X is inbred. By replacing in Eq. (S2.1) with (S2.2) and adding the *residual* *breeding value* (RBV) $\phi_{X}^{*} = \delta_{S} + \delta_{D},$ we arrive at the animal model, or “parental regression”, already discussed in Searle [58]:

$a_{*X} = 0.5 a_{*S} + 0.5 a_{*D} + \phi_{X}^{*}$ (S2.3)

This expression relates the BV of equally contributing parents to their offspring’s BV (see also Bulmer [3]). Under a Gaussian distribution of BV, Eq. (S1.3) is an example of a recursive *causal model* (see page 27 in [4]) in which the BV are *caused* by parental BV that are transmitted in equal proportion. The “noisy” RBV reflects the uncertainty in predicting *a*_X_ caused Mendelian segregation [56]. Each RBV is *unique* because it is likely that meioses result in segregation events that are all different, for every animal within a full-sib family. In a linear model sense, the *regressors* are both parental BV with nonzero standardized partial regression coefficients or *path coefficients* (see Supplementary Information 1, section *Lineages*, *pedigree, graphs, and breeding values*). In the “parental regression”, path coefficients are always equal to 0.5 regardless of the level of inbreeding of either the parents or the progeny (Foulley and Chevalet [59]). Interestingly, Eq. (S1.3) represents an autonomous process of “BV generation” that involves *individual* mechanisms (segregation, recombination, linkage, linkage disequilibrium), or *population* factors (selection, nonrandom union of gametes, non-random matings) that determine the value of *a_*_*_X_ (see expression (1.41) in [4]) and that does not depend on the RBV of the descendants of X.

Because the residuals in a Gaussian causal model such as Eq. (S2.3) are mutually independent, the distribution of BV possess the Markov property [see Additional file 1]), which in turn allows the covariance between the BV of the parents and offspring to be *recursive*. Consequently, the multivariate distribution of BV from a pedigree is determined uniquely by the distribution of the RBV [4, 5].

***Identity disequilibrium due to recombination at a large number of sites***

Weir and Cockerham [7] defined *identity disequilibrium* as the following difference:

$P\left( X_{1}\equiv Y_{1},X_{2}\equiv Y_{2} \right)-P\left( X_{1}\equiv Y_{1} \right) P\left( X_{2}\equiv Y_{2} \right)=P\left( X_{1}\equiv Y_{1},X_{2}\equiv Y_{2} \right)-P\left( X\equiv Y \right)^{2}$ (S2.4)

The second term results from the *P*(IBD) being equal at all sites under independent loci. Identity disequilibrium increases with inbreeding (Weir and Cockerham [6]) and linkage (Tachida and Cockerham [60]). A generalization of identity disequilibrium to a large number of sites can be found in the theorem 5.1 of Esary et al [61], which says that a pair of multivariate binary random variables with *non-negative covariance* among them are *associated* and display *positive quadrant dependence* [13]. As long as the covariance between BV is non-negative, the theorem says that the *P*(IBD) from *n* sites is always greater or at least equal to the *P*(IBD) at *n* – 1 sites taken together, which in turn is greater than the *P*(IBD) at *n* – 2 sites taken together and so on, up to the single locus *P*(IBD). This property of the *joint distribution* of IBD implies that BV are *associated* random variables. Under gametic equilibrium, this is easy to prove because cov(*a*_X_, *a*_Y_) = **A**$\sigma_{A}^{2}$ and, by definition, both terms are non-negative. Therefore, the *degree of relatedness* is always non-negative and the cov(*a*_X_, *a*_Y_) ≥ 0 leads to *positive quadrant dependence* (Lehmann [13]). Due to *positive quadrant dependence*, animals with larger BV than the mean will tend to have progeny with larger BV than the mean, whereas animals with smaller BV than the mean will produce progeny that tend to have smaller BV than the mean.

Guo [22] defined the fraction of the genome that is shared IBD between two relatives as a *continuous* version of *P*(IBD). For the relationship between a grandsire and a grand-progeny, the estimator of *P*(IBD) by Guo [22] is equal to the parameter *P*_G_ of Veller et al [62], whereas the estimator of *P*(IBD) for the relationship between the grand-dam and her grand-progeny is equal to 1 − *P*_G_. Here, identity disequilibrium due to differential ancestral recombination is defined as the difference between the *observed* value of the estimated contribution of the sire of parent G, $\hat{P}_{G}$, with respect to its *expectation* under gametic equilibrium, $\hat{P}_{G}$− E$\left( \hat{P}_{G} \right)$ that is equal to $\hat{P}_{G}$− 0.50. Only when the identity disequilibrium is zero, is the *P*(IBD) in Eq. (S2.1) equal to *P*(X ≡ Y), whereas for any other value than $\hat{P}_{G}$= 0.5, the *P*(IBD) is equal to the joint distribution to the left of Eq. (S2.1).

***Breeding values under identity disequilibrium***

Modelling gametic effects under identity disequilibrium requires additional terms in Eq. (S2.1), which are then equal to:

$g_{S_{X}} = 0.5 \left( a_{*S} + \varepsilon_{S} \right) + \delta_{S} g_{D_{X}}= 0.5 \left( a_{*D} + \varepsilon_{D} \right) + \delta_{D}$. (S2.5)

The random variable ε_S_ represents the amount of identity disequilibrium in the gamete from the sire, and ε_D_ is the amount of identity disequilibrium from the dam. Adding and subtracting to the BV of X the gametic effects in Eq. (S2.5) results in

$a_{X}= 0.5 \left( a_{*S}+\varepsilon_{S} \right)+0.5\left( a_{*D}+\varepsilon_{D} \right)+\left( a_{X}-0.5\left( a_{*S}+\varepsilon_{S} \right)-0.5\left( a_{*D}+\varepsilon_{D} \right) \right)$.

The term between parentheses is the RBV under identity disequilibrium and equal to:

$\phi_{X}=a_{X} -0.5 \left( a_{*S}+ \varepsilon_{S} \right)-0.5 \left( a_{*D}+\varepsilon_{D} \right)$, (S2.6)

and the causal model for breeding value under identity disequilibrium is:

$a_{X}=0.5 \left( a_{*S}+\varepsilon_{S} \right)+0.5\left( a_{*D}+\varepsilon_{D} \right) + \phi_{X}$. (S2.7)

Our next task is to associate the random variables ε_S_ and ε_D_ to the parameters *P*_S_ and *P*_D_, respectively. In doing that we write SS for the paternal grandsire, DS for the maternal grandam, SD for the maternal grandsire and DD for the maternal granddam of animal X. Following an idea of Wang and Xu [63] who expressed BV as linear combinations of grandparental BV, we set identity disequilibrium terms to be equal to:

$\varepsilon_{S}=P_{S} a_{\mathrm{SS}}+\left( 1-P_{S} \right) a_{\mathrm{DS}}-0.5\left( a_{\mathrm{SS}}+a_{\mathrm{DS}} \right)$, (S2.8)

and

$\varepsilon_{D}=P_{D} a_{\mathrm{SD}}+\left( 1-P_{D} \right) a_{\mathrm{DD}}-0.5 \left( a_{\mathrm{SD}}+a_{\mathrm{DD}} \right)$. (S2.9)

Further algebraic work in Eq. (S2.8) results in:

$\varepsilon_{S} =\left( P_{S}-0.5 \right) a_{\mathrm{SS}}+\left( 1-P_{S}- 0.5 \right) a_{\mathrm{DS}}=\left( P_{S}-0.5 \right) a_{\mathrm{SS}}-\left( P_{S}-0.5 \right) a_{\mathrm{DS}}$,

which leads to:

$\varepsilon_{S}=\left( P_{S}-0.5 \right) \left( a_{\mathrm{SS}}-a_{\mathrm{DS}} \right)$. (S2.10)

The corresponding expression for the maternal identity disequilibrium term is:

$\varepsilon_{D}=\left( P_{D}-0.5 \right) \left( a_{\mathrm{SD}}-a_{\mathrm{DD}} \right)$. (S2.11)

We now reparametrize the “gametic survival probabilities” *P*_S_ and *P*_D_ deviated from their expectation to obtain:

$0.5 \left( P_{S}- 0.5 \right)=\beta_{S} 0.5 \left( P_{D}-0.5 \right)=\beta_{D}$. (S2.12)

A check of the new parameters shows that β_S_ = 0 = β_D_ under identity equilibrium when *P*_S_ = 0.5 = *P*_D_. In the limiting case that *P*_S_ or *P*_D_ are equal to 1, inspection of Eq. (S2.12) leads to β = 0.25. Conversely, if *P*_S_ or *P*_D_ are equal to 0, β = −0.25. Hence, the parameter space of β is −0.25 ≤ β_S_, β_D_ ≤ 0.25.

After replacing in Eq. (S2.7) with Eqs. (S2.10), (S2.11), and (S2.12) results in the following recursive equation for BV, which is the ancestral regression of Cantet et al [8]:

$a_{X} =0.5 a_{S} +0.5 a_{D} +\beta_{S}\left( a_{\mathrm{SS}} -a_{\mathrm{DS}} \right)+ \beta_{D}\left( a_{\mathrm{SD}} - a_{\mathrm{DD}} \right)+\phi_{X}$. (S2.13)

Letting PGP = 0.5(*a*_SS_ – *a*_DS_) and MGP = 0.5(*a*_SD_ – *a*_DD_), β_S_ and β_D_ are equal to the following path coefficients:

$\beta_{S}= \frac{\Sigma_{X,PGP}-\Sigma_{PGP,R_{S}} \Sigma_{R_{S}}^{-1} \Sigma_{R_{S,X}}}{\Sigma_{\mathrm{PGP}}-\Sigma_{PGP,R_{S}}\Sigma_{R_{S}}^{-1} \Sigma_{R_{S},PGP}} \beta_{D}= \frac{\Sigma_{X,MGP}-\Sigma_{MGP,R_{D}} \Sigma_{R_{D}}^{-1} \Sigma_{R_{D,X}}}{\Sigma_{\mathrm{MGP}}-\Sigma_{MGP,R_{D}}\Sigma_{R_{D}}^{-1} \Sigma_{R_{D},MGP}}$ . (S2.14)

where $\boldsymbol{\Sigma}_{ij}=cov\left( a_{i}, a_{j} \right)$ are the elements of the covariance matrix of BV under AR, **Σ**. Figure S2 displays a DAG of the AR and S3 shows the AMG representation of the PAR for the same animal. The set R_S_ for β_S_ includes the BV [*a*_SD_, *a*_DD_, *a*_S_, *a*_D_] such that the covariance matrix $\boldsymbol{\Sigma}_{R_{S}}$ is 4×4. Similarly, when defining β_D_ the set R_D_ includes the BV [*a*_SS_, *a*_DS_, *a*_S_, *a*_D_] with covariance matrix $\boldsymbol{\Sigma}_{R_{D}}$.

**Additional file 2 Figure S3** **DAG representation of ancestral regression (AR)**

**
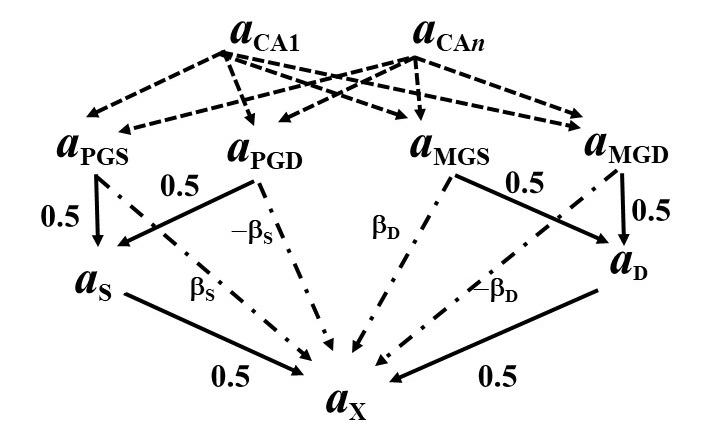
**

**Additional file 2 Figure S4** **Acyclic mixed graph representation of ancestral regression to parents (PAR)**

**
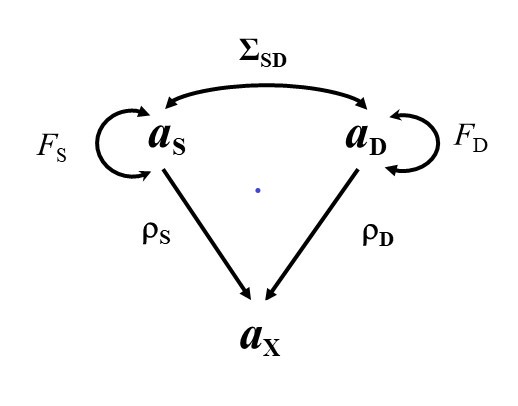
**

***Multivariate normality of breeding values under disequilibrium***

A general central limit theorem for *associated* random variables under very mild requirements was derived by Cox and Grimmett [64]: if $\sigma_{A}^{2} > 0$ and $\mathrm{cov}\left( a_{X}, a_{Y} \right)\to0$ as the number of sites (*M*), grows large, then the BV are asymptotically multivariate normal. By a seemingly independent argument to the previous result and using asymptotic theory for *M* *positive quadrant dependent* random variables, Tallis [65] proved that, when causal genes are linked, the distribution of BV remains multivariate normal.
