# Supplementary material for: Causal inference for the covariance between breeding values under identity disequilibrium
Source: Genet Sel Evol. 2022 Sep 23;54:64. doi: 10.1186/s12711-022-00750-6 (PMC9502921; doi:10.1186/s12711-022-00750-6)
Supplement: Supplementary file 3 — Additional file 3. Covariance matrix of breeding values under identity disequilibrium [66–68]. [file 12711_2022_750_MOESM3_ESM.docx]

**Additional file 3**

**Covariance matrix of BV under identity disequilibrium**

By definition, the covariance between the breeding value (BV) of an ancestor and a descendant is equal to:

$\mathrm{cov}\left( a_{A}, a_{X} \right)= E\left( a_{A} a_{X} \right)- E\left( a_{A} \right) E\left( a_{Y} \right)$.

Given that the expected value of any BV is zero, only the expectation of the product needs to be obtained, which can be calculated by the double expectation theorem (Bickel and Doksum [66]) conditional in the distribution of the lineage:

$E\left( a_{A} a_{X} \right) = E\left[ E\left( a_{A} a_{X}\left| a_{A}\to...\to a_{X} \right. \right) \right]$.

In a DAG [see Additional file 2 Figure S3] or in an AMG [see Additional file 2 Figure S4], a *directed* path (Fox et al [39]) goes from the BV of A to the BV of one of its progeny, and eventually ends up in the BV of its descendant X, i.e., *a*_A_ →…→ *a*_X_, graphically expressing that a fraction of the genome of the *ancestor* may still survive in the genome of X. Now, consider the given meiosis by which the parent in the lineage passed to X the gamete with effect $g_{P_{X}}$. Out of all possible values of $a_{P_{X}}$over the inference space, the gametes carries $a_{*PX}+\beta_{P_{X}} \left( a_{S, \mathrm{PX}} -a_{D, \mathrm{PX}} \right)$, such that the resulting distribution has a narrower inference space than the distribution of $a_{*PX}$. This is precisely an *intervention* in the language of Pearl [4, page 70] in which the IBD between X and its grandparent is a function of the single value of the path coefficient $\beta_{P_{X}}$ such that the double expectation under Pearl’s [4] *do*-operator is equal to:

$$E\left[ a_{A}, a_{X}\left| do\left( g_{P_{X}}\to0.5 \left[ a_{\mathrm{PX}}+\beta_{P_{X}} \left( a_{S, \mathrm{PX}}- a_{D, \mathrm{PX}} \right) \right] \right) \right. \right]$$

$= \mathrm{cov}\left( a_{A}, a_{X}\left| a_{X} =0.5 a_{S}+0.5 a_{D}+\beta_{S} \left( a_{\mathrm{SS}}-a_{\mathrm{DS}} \right)+\beta_{D} \left( a_{\mathrm{SD}}-a_{\mathrm{DD}} \right) \right. \right)$.

Finally,

$\mathrm{cov}\left( a_{A}, a_{X} \right)=\mathrm{cov}\left( a_{A}, 0.5 a_{S}+0.5 a_{D}+\beta_{S} \left( a_{\mathrm{SS}}-a_{\mathrm{DS}} \right)+\beta_{D} \left( a_{\mathrm{SD}}-a_{\mathrm{DD}} \right) \right)$.

***A recursive calculation of the rows and columns of*** ***Σ***

For a pedigree involving the breeding values of *q* animals, let **Σ** be the covariance matrix of AR (see Equation (10) in Cantet et al [8]). The expression and estimation of the elements of **Σ** is facilitated if we write down the rows of **B**. For animal X, this is a 1×*q* vector (**B**_X_) having most elements equal to zero except for six non-zero path coefficients that relate the BV of X to those of its parents and grandparents. Ordering arbitrarily these path coefficients as belonging to SS, DS, SD, DD, S and D, **B**_X_ is as follows:

$\mathbf{B}_{X}= \left[ 0 ..0..\beta_{S}.. {-\beta}_{S} .. \beta_{D} ..{-\beta}_{D} .. 0.5 .. 0.5 .. \right].$ (S3.1)

To estimate β_S_*_i_* and β_D_*_i_* for animal *i*, it is useful to consider a version of **B**_X_ without the zeros, say **B**_X_*_r_*, which is equal to:

$\mathbf{B}_{X,r}=\left[ \beta_{S} {-\beta}_{S} \beta_{D} {-\beta}_{D} 0.5 0.5 \right]$. (S3.2)

***Markov property and recursion***

In the AR, grandparents are the “founder generation”. Vigeland [16] proved that any genetic relationship is *constructible* using any founder generation as long as the inbreeding coefficients of the founders and all relationships among them are correctly specified. The set of BV adjacent to an individual formed by parents, progeny, and mates, contain the *locally* *sufficient statistics* [5] to estimate β_S_ and β_D_, and is called a “Markov blanket” [34]. However, estimation of β_S_ and β_D_ using progeny genotypes is quite involved because covariances include the identity disequilibrium parameters β of the offspring in a non-linear fashion. Hence, we form the Markov blanket with *locally* sufficient statistics: the BV of grandparents and parents of X in vector $\boldsymbol{a}_{A}'= \left[ a_{\mathrm{SS}}, a_{\mathrm{DS}},a_{\mathrm{SD}},a_{\mathrm{DD}},a_{S},a_{D} \right]$ with covariance matrix equal to:

$\mathrm{Var}\left( \boldsymbol{a}_{A} \right) = \boldsymbol{\Sigma}_{A}= \left[ \begin{matrix} \boldsymbol{\Sigma}_{SS,SS} & \boldsymbol{\Sigma}_{SS,DS} & \boldsymbol{\Sigma}_{SS,SD} & \boldsymbol{\Sigma}_{SS,DD} & \boldsymbol{\Sigma}_{SS,S} & \boldsymbol{\Sigma}_{SS,D} \\ \boldsymbol{\Sigma}_{DS,SS} & \boldsymbol{\Sigma}_{DS,DS} & \boldsymbol{\Sigma}_{DS,SD} & \boldsymbol{\Sigma}_{DS,DD} & \boldsymbol{\Sigma}_{DS,S} & \boldsymbol{\Sigma}_{DS,D} \\ \boldsymbol{\Sigma}_{SD,SS} & \boldsymbol{\Sigma}_{SD,DS} & \boldsymbol{\Sigma}_{SD,SD} & \boldsymbol{\Sigma}_{SD,DD} & \boldsymbol{\Sigma}_{SD,S} & \boldsymbol{\Sigma}_{SD,D} \\ \boldsymbol{\Sigma}_{DD,SS} & \boldsymbol{\Sigma}_{DD,DS} & \boldsymbol{\Sigma}_{DD,SD} & \boldsymbol{\Sigma}_{DD,DD} & \boldsymbol{\Sigma}_{DD,S} & \boldsymbol{\Sigma}_{DD,D} \\ \boldsymbol{\Sigma}_{S,SS} & \boldsymbol{\Sigma}_{S,DS} & \boldsymbol{\Sigma}_{S,SD} & \boldsymbol{\Sigma}_{S,DD} & \boldsymbol{\Sigma}_{S,S} & \boldsymbol{\Sigma}_{S,D} \\ \boldsymbol{\Sigma}_{D,SS} & \boldsymbol{\Sigma}_{D,DS} & \boldsymbol{\Sigma}_{D,SD} & \boldsymbol{\Sigma}_{D,DD} & \boldsymbol{\Sigma}_{D,S} & \boldsymbol{\Sigma}_{D,D} \end{matrix} \right]$. (S3.3)

Under multivariate normality of BV, the parental Markov property allows for a *recursive factorization* (Andersson and Perlman [67]) of the distribution of BV *f*(***a***) as a product of the conditional distributions of *a_i_* given its Markov blanket ***a***_A_*_i_*:

$$f\left( \boldsymbol{a} \right)=f\left( a_{SS_{i}}, a_{DS_{i}}, a_{SD_{i}}, a_{DD_{i}}, a_{S_{i}}, a_{D_{i}}, a_{i} \right)$$

$= \prod_{i} f\left( a_{i}\left| a_{SS_{i}}, a_{DS_{i}}, a_{SD_{i}}, a_{DD_{i}}, a_{S_{i}}, a_{D_{i}} \right. \right) = \prod_{i} f\left( a_{i}\left| \boldsymbol{a}_{A_{i}} \right. \right)$ . (S3.4)

The factorization sets operational the following recursion of *a*_X_ in ***a***_AX_:

$a_{X} = \mathbf{B}_{X r}' \boldsymbol{a}_{A_{X}} + \phi_{X}$ (S3.5)

Any estimation algorithm of **Σ** involves the 6 × 1 vector:

$\mathrm{cov}\left( \boldsymbol{a}_{A_{X}},a_{X} \right)\mathbf{=}\left[ \begin{matrix} \Sigma_{SS,X} & \Sigma_{DS,X} & \Sigma_{SD,X} & \Sigma_{DD,X} & \Sigma_{S,X} & \Sigma_{D,X} \end{matrix} \right] ' = \boldsymbol{\Sigma}_{X A}$. (S3.6)

By post-multiplying the transpose of $\mathbf{B}_{X r}$ to $\boldsymbol{\Sigma}_{A}$, we obtain that $\boldsymbol{\Sigma}_{A} \mathbf{B}_{X r}^{'}= \boldsymbol{\Sigma}_{X A}$. Now, we are able to write a 7×7 covariance matrix that has a similar structure to that of a Gaussian regression process with noise (see expressions (2.20) to (2.24) on page 16 in Rasmussen and Williams [68]) as follows:

$\mathrm{Var}\left[ \begin{matrix} \boldsymbol{a}_{A} \\ a_{X} \end{matrix} \right] = \left[ \begin{matrix} \boldsymbol{\Sigma}_{A} & \boldsymbol{\Sigma}_{A X} \\ \boldsymbol{\Sigma}_{A X} & \boldsymbol{\Sigma}_{X} \end{matrix} \right] = \left[ \begin{matrix} \boldsymbol{\Sigma}_{A} & \boldsymbol{\Sigma}_{A} \mathbf{B}_{X r}' \\ \mathbf{B}_{X r} \boldsymbol{\Sigma}_{A} & \mathbf{B}_{X r} \boldsymbol{\Sigma}_{A} \mathbf{B}_{X r}' + \mathrm{Var}\left( \phi_{\mathrm{AR}\left( X \right)} \right) \end{matrix} \right]$. (S3.7)

However, since the only parameters in Eq. (S3.7) are β_S_ and β_D_, we have to reduce the size of the vector of unknowns from 6 to 2, and this is done below.

***Estimating equations for* β_S_** ***and*** **β_D_**

Begin by collecting the covariance between the individual and its grandparents and parents:

$$\Sigma_{SS,X} = 0.5 \Sigma_{SS,S}+0.5 \Sigma_{SS,D}+\beta_{S} \left( 1+F_{\mathrm{SS}}-\Sigma_{SS,DS} \right)+ \beta_{D} \left( \Sigma_{SS,SD}-\Sigma_{SS,DD} \right)$$

$$\Sigma_{DS,X} =0.5 \Sigma_{DS,S} +0.5 \Sigma_{DS,D}-\beta_{S} \left( 1+F_{\mathrm{DS}}-\Sigma_{SS,DS} \right)+\beta_{D} \left( \Sigma_{DS,SD}-\Sigma_{DS,DD} \right)$$

$$\Sigma_{SD,X} =0.5 \Sigma_{SD,S} +0.5 \Sigma_{SD,D}+\beta_{S} \left( \Sigma_{SD,SS}- \Sigma_{SD,DS} \right)+\beta_{D} \left( 1+F_{\mathrm{SD}}- \Sigma_{SD,DD} \right)$$

$$\Sigma_{DD,X}=0.5 \Sigma_{DD,S}+0.5 \Sigma_{DD,D}+\beta_{S} \left( \Sigma_{DD,SS}-\Sigma_{DD,DS} \right)- \beta_{D} \left( 1+F_{\mathrm{DD}}-\Sigma_{SD,DD} \right)$$

$$\Sigma_{S, X} = 0.5 \left( 1 + F_{S}+ \Sigma_{S,D} \right) + \beta_{S} \left( \Sigma_{SS,S} - \Sigma_{DS,S} \right)+ \beta_{D} \left( \Sigma_{SD,S}- \Sigma_{DD,S} \right)$$

$\Sigma_{D, X} = 0.5 \left( 1 + F_{D}+ \Sigma_{S,D} \right) + \beta_{S} \left( \Sigma_{SS,D}- \Sigma_{DS,D} \right)+ \beta_{D} \left( \Sigma_{SD,D}- \Sigma_{DD,D} \right)$.

Now, we rearrange them as follows:

$$\left( 1+ F_{S S}- \Sigma_{SS, \mathrm{DS}} \right) \beta_{s} + \left( \Sigma_{SS, \mathrm{SD}} - \Sigma_{\mathrm{SS} ,DD} \right) \beta_{D}=\Sigma_{S S ,X}-0.5 \left( \Sigma_{SS, S}+\Sigma_{SS, D} \right)$$

$$-\left( 1+F_{D S}- \Sigma_{SS, \mathrm{DS}} \right) \beta_{s}+\left( \Sigma_{DS, \mathrm{SD}}- \Sigma_{DS, \mathrm{DD}} \right) \beta_{D}=\Sigma_{\mathrm{DS} ,X}-0.5 \left( \Sigma_{DS, S}+ \Sigma_{DS, D} \right)$$

$$\left( \Sigma_{SS, \mathrm{SD}} - \Sigma_{DS, \mathrm{SD}} \right) \beta_{s} + \left( 1+ F_{S D}- \Sigma_{SD,DD} \right) \beta_{D} = \Sigma_{SD,X}- 0.5 \left( \Sigma_{SD,S}+\Sigma_{SD,D} \right)$$

$$\left( \Sigma_{\mathrm{SS} ,DD}- \Sigma_{DS, \mathrm{DD}} \right) \beta_{s}-\left( 1+ F_{D D}- \Sigma_{SD,DD} \right) \beta_{D} = \Sigma_{\mathrm{DD} ,X}- 0.5 \left( \Sigma_{DD, S}+ \Sigma_{DD, D} \right)$$

$$\left( \Sigma_{SS,S} - \Sigma_{DS,S} \right) \beta_{S} + \left( \Sigma_{SD,S}- \Sigma_{DD,S} \right) \beta_{D} = \Sigma_{S, X} -0.5 \left( 1+F_{S}+\Sigma_{S,D} \right)$$

$\left( \Sigma_{SS,D}- \Sigma_{DS,D} \right) \beta_{S} + \left( \Sigma_{SD,D} - \Sigma_{DD,D} \right) \beta_{D} = \Sigma_{D,X}-0.5 \left( 1+F_{D}+\Sigma_{S,D} \right)$.

In matrix notation, these equations look like this:

$\left[ \begin{aligned} \left( 1+ F_{S S}- \Sigma_{SS, \mathrm{DS}} \right) \left( \Sigma_{SS, \mathrm{SD}} - \Sigma_{\mathrm{SS} ,DD} \right) \\ -\left( 1+F_{D S}- \Sigma_{SS, \mathrm{DS}} \right) \left( \Sigma_{DS, \mathrm{SD}}- \Sigma_{DS, \mathrm{DD}} \right) \\ \left( \Sigma_{SS, \mathrm{SD}} - \Sigma_{DS, \mathrm{SD}} \right) \left( 1+ F_{S D}- \Sigma_{SD,DD} \right) \\ \left( \Sigma_{SS,DD}-\Sigma_{DS, \mathrm{DD}} \right) \beta_{s} -\left( 1+F_{\mathrm{DD}}-\Sigma_{SD,DD} \right) \\ \left( \Sigma_{SS,S} - \Sigma_{DS,S} \right) \beta_{S} \left( \Sigma_{SD,S}- \Sigma_{DD,S} \right) \\ \left( \Sigma_{SS,D}- \Sigma_{DS,D} \right) \beta_{S} \left( \Sigma_{SD,D} - \Sigma_{DD,D} \right) \end{aligned} \right]\left[ \begin{aligned} \beta_{s} \\ \beta_{D} \end{aligned} \right]=\left[ \begin{aligned} \Sigma_{\mathrm{SS} ,X}-0.5 \left( \Sigma_{SS, S}+\Sigma_{SS, D} \right) \\ \Sigma_{\mathrm{DS} ,X}-0.5 \left( \Sigma_{DS, S}+\Sigma_{DS, D} \right) \\ \Sigma_{SD,X}-0.5 \left( \Sigma_{SD,S}+\Sigma_{SD,D} \right) \\ \Sigma_{DD,X}-0.5 \left( \Sigma_{DD, S}+\Sigma_{DD, D} \right) \\ \Sigma_{S, X} -0.5 \left( 1+F_{S}+\Sigma_{S,D} \right) \\ \Sigma_{D,X}-0.5 \left( 1+F_{D}+\Sigma_{S,D} \right) \end{aligned} \right]$.

Now, on defining:

$\boldsymbol{L} = \left[ \begin{matrix} 1 & 0 \\ -1 & 0 \\ 0 & 1 \\ 0 & -1 \\ \frac{1}{2 \beta_{S}} & 0 \\ 0 & \frac{1}{2 \beta_{D}} \end{matrix} \right] \boldsymbol{\beta} = \left[ \begin{matrix} \beta_{S} \\ \beta_{D} \end{matrix} \right]$,

the equations can be written more succinctly as$\left( \boldsymbol{\Sigma}_{A}^{0.5} \mathbf{L} \right) \boldsymbol{\beta} = \boldsymbol{\Sigma}_{A,X}$. Then, use of least squares results in the two equation systems $\left( \mathbf{L} ' \boldsymbol{\Sigma}_{A}^{0.5} ' \right) \left( \boldsymbol{\Sigma}_{A}^{0.5} \mathbf{L} \right) \boldsymbol{\beta} = \mathbf{L} ' \boldsymbol{\Sigma}_{A,X}$. After much and tedious algebra, the elements are the following:

$\begin{matrix} \left( \mathbf{L} ' \boldsymbol{\Sigma}_{A} \mathbf{L} \right)_{11} = \left( 1+F_{\mathrm{SS}}-\Sigma_{SS, \mathrm{DS}} \right)^{2}+\left( 1+ F_{\mathrm{DS}}-\Sigma_{SS, \mathrm{DS}} \right)^{2}+\left( \Sigma_{SS, \mathrm{SD}}-\Sigma_{DS, \mathrm{SD}} \right)^{2} \\ +\left( \Sigma_{\mathrm{SS} ,DD}-\Sigma_{DS, \mathrm{DD}} \right)^{2}+\left( \Sigma_{SS,S} -\Sigma_{DS,S} \right)^{2}+\left( \Sigma_{SS,D}- \Sigma_{DS,D} \right)^{2} \end{matrix}$,

$$\begin{matrix} \left( \mathbf{L} ' \boldsymbol{\Sigma}_{A} \mathbf{L} \right)_{12}=\left( 1+F_{\mathrm{SS}}- \Sigma_{SS, \mathrm{DS}} \right)\left( \Sigma_{SS, \mathrm{SD}}-\Sigma_{\mathrm{SS} ,DD} \right)-\left( 1+F_{\mathrm{DS}}-\Sigma_{SS, \mathrm{DS}} \right)\left( \Sigma_{DS, \mathrm{SD}}-\Sigma_{DS, \mathrm{DD}} \right) \\ +\left( \Sigma_{SS, \mathrm{SD}} - \Sigma_{DS, \mathrm{SD}} \right)\left( 1+ F_{\mathrm{SD}}- \Sigma_{SD,DD} \right)-\left( \Sigma_{\mathrm{SS} ,DD}- \Sigma_{DS, \mathrm{DD}} \right)\left( 1+F_{D D}-\Sigma_{SD,DD} \right) \\ +\left( \Sigma_{SS,S} - \Sigma_{DS,S} \right)\left( \Sigma_{SD,S}- \Sigma_{DD,S} \right)+ \left( \Sigma_{SS,D}- \Sigma_{DS,D} \right)\left( \Sigma_{SD,D}- \Sigma_{DD,D} \right) \end{matrix}$$

$\begin{matrix} \left( \mathbf{L} ' \boldsymbol{\Sigma}_{A} \mathbf{L} \right)_{22} = \left( \Sigma_{SS, \mathrm{SD}} - \Sigma_{\mathrm{SS} ,DD} \right)^{2}+\left( \Sigma_{DS, \mathrm{SD}}- \Sigma_{DS, \mathrm{DD}} \right)^{2}+\left( 1+ F_{S D}- \Sigma_{SD,DD} \right)^{2} \\ + \left( 1+ F_{D D}- \Sigma_{SD,DD} \right)^{2}+\left( \Sigma_{SD,S}- \Sigma_{DD,S} \right)^{2}+\left( \Sigma_{SD,D} - \Sigma_{DD,D} \right)^{2} \end{matrix}$,

$$\begin{matrix} \left( \mathbf{L} ' \boldsymbol{\Sigma}_{A,X} \right)_{1}=\left( 1+F_{S S}- \Sigma_{SS, \mathrm{DS}} \right)\left( \Sigma_{SS, \mathrm{SD}}-\Sigma_{\mathrm{SS} ,DD} \right)-\left( 1+F_{\mathrm{DS}}- \Sigma_{SS, \mathrm{DS}} \right)\left( \Sigma_{DS, \mathrm{SD}}-\Sigma_{DS, \mathrm{DD}} \right) \\ +\left( \Sigma_{SS, \mathrm{SD}} - \Sigma_{DS, \mathrm{SD}} \right)\left( 1+ F_{S D}- \Sigma_{SD,DD} \right)-\left( \Sigma_{\mathrm{SS} ,DD}- \Sigma_{DS, \mathrm{DD}} \right)\left( 1+ F_{D D}- \Sigma_{SD,DD} \right) \\ +\left( \Sigma_{SS,S} - \Sigma_{DS,S} \right)\left( \Sigma_{SD,S}- \Sigma_{DD,S} \right)+ \left( \Sigma_{SS,D}- \Sigma_{DS,D} \right)\left( \Sigma_{SD,D} - \Sigma_{DD,D} \right) \end{matrix},$$

$\left( \mathbf{L}^{\mathbf{'}}\boldsymbol{\Sigma}_{A,X} \right)_{2} = \left( \Sigma_{SS, \mathrm{SD}} - \Sigma_{\mathrm{SS} ,DD} \right)\left( c_{S S ,X}- 0.5 \left( \Sigma_{SS, S}+ \Sigma_{SS, D} \right) \right)+$

$$\left( \Sigma_{DS, \mathrm{SD}}- \Sigma_{DS, \mathrm{DD}} \right)\left( c_{\mathrm{DS} ,X}- 0.5 \left( \Sigma_{DS, S}+ \Sigma_{DS, D} \right) \right)$$

$$\left( c_{\mathrm{DS} ,X}- 0.5 \left( \Sigma_{DS, S}+ \Sigma_{DS, D} \right) \right) +\left( 1+ F_{S D}- \Sigma_{SD,DD} \right)\left( c_{\mathrm{SD} ,X}- 0.5 \left( \Sigma_{SD,S}+\Sigma_{SD,D} \right) \right)- \left( 1+ F_{\mathrm{DD}}- \Sigma_{SD,DD} \right)\left( c_{\mathrm{DD} ,X}- 0.5 \left( \Sigma_{DD, S}+ \Sigma_{DD, D} \right) \right)$$

$$+\left( \Sigma_{SD,S}-\Sigma_{DD,S} \right)\left( c_{S, X}-0.5 \left( 1+F_{S}+ \Sigma_{S,D} \right) \right)\left( \Sigma_{SD,D}-\Sigma_{DD,D} \right)$$

$$\left( c_{D,X} -0.5 \left( 1+F_{D}+\Sigma_{S,D} \right) \right)$$
